# Development of an Evidence-Based, Theory-Informed National Survey of Physician Preparedness for Genomic Medicine and Preferences for Genomics Continuing Education

**DOI:** 10.3389/fgene.2020.00059

**Published:** 2020-03-03

**Authors:** Belinda J. McClaren, Emily A. King, Erin Crellin, Clara Gaff, Sylvia A. Metcalfe, Amy Nisselle

**Affiliations:** ^1^ Australian Genomics Health Alliance, Melbourne, VIC, Australia; ^2^ Genomics in Society, Murdoch Children’s Research Institute, Melbourne, VIC, Australia; ^3^ Department of Pediatrics, The University of Melbourne, Melbourne, VIC, Australia

**Keywords:** survey development, genomic education, qualitative, Delphi, theory

## Abstract

Despite some early implementation of genomic medicine globally, there is a lack of rigorous, large-scale assessments of medical specialists' current practice and continuing education needs. As a first step to addressing this gap, we describe the development of a robust, expert-reviewed, survey using a mixed-methods sequential study design. We conducted semi-structured qualitative interviews with 32 education providers and 86 non-genetic medical specialists about current genomic medicine practice and need for continuing education. Key concepts were identified and used as an initial framework for the survey. These were: personal characteristics (medical specialty, years of practice); current practice of genomics in clinical and research settings; perception of how proximal genomic medicine is to practice; perception of preparedness (competence and confidence); and, preferences for future roles and models of care in genomic medicine and for continuing education. Potential survey questions that related to at least one of these concepts were identified from the literature or were created if no suitable question existed. Using a modified, reactive Delphi approach, questions were reviewed by a panel of 22 experts. Experts were selected purposefully representing four areas of expertise: non-genetic medical specialties; clinical genetics; genetic/genomic education and evaluation; and implementation science. Three Delphi rounds assessed relevance, clarity and importance of each question. The questions were also mapped to the behaviour change wheel theoretical framework which encompasses capability, opportunity and motivation (COM-B). The survey (included as supplementary material) was then tested with a small group of non-genetic medical specialists and feedback was written or verbal in ‘talk-aloud', cognitive interviews. The final survey was then piloted with a further 29 specialists. We describe the methodology to create a robust, data- and theory-informed survey. The final survey captures not only levels of experience, practice of genomics and preferences for education but also the challenges around engaging with education. Survey data will provide evidence for education providers to inform development of education which meets learner needs and contributes to a medical workforce that is literate in genomics and more confident to competently practice genomic medicine.

## Introduction

Genomic medicine is increasingly present in clinical practice, requiring non-genetic medical specialists to ‘develop and expand' expertise (Burton, 2011; Burton et al., 2017; Gaff et al., 2017). As the growing use of genomic investigations is rapidly exceeding the capacity of the clinical genetics workforce (Slade et al., 2016; Maiese et al., 2019), different approaches to the practice of genomic medicine will be needed. Consequently, it is likely non-genetic medical specialists will need to alter their current practices and behaviors to incorporate genomic medicine, with some taking on tasks previously in the remit of genetic health professionals (Bowdin et al., 2016; Ormond et al., 2019). This may include directly requesting tests for patients, and discussing results, rather than referring to a clinical genetic service.

Education has been suggested as an approach to address gaps in skills and confidence of non-genetic medical specialists to practice genomic medicine (Feero and Green, 2011; Paul et al., 2018; Crellin et al., 2019). To date, there has been no systematic approach to measure the educational needs of the medical workforce on a national scale in Australia, and to understand how these needs may differ across diverse disciplines. Therefore, there is little evidence available to inform the design and development of system-wide educational or training activities to support non-genetic medical specialists in acquiring the skills, confidence and competence they need to appropriately integrate genomic medicine into their clinical practice. The implementation of genomics in healthcare is being addressed at a national level in a number of countries (Stark et al., 2019a). For instance, in Australia the Federal Government has developed a National Genomics Health Policy Framework that identifies genomic literacy of health professionals as a national priority (Australian Government Department of Health, 2017). For countries with a publicly funded (socialized) health system, a health system-wide approach to understanding the practice and needs of diverse disciplines can enable government decision-making on how investment in education and training may best be deployed. Capturing details of current practice, perceptions of future practice and preferences for learning can also provide much needed evidence for education providers about the areas on which to focus their efforts and resources. For example, are there particular sub-specialties of medicine for whom the need and desire for educational activities in genomics is greatest? Are there other specialties in which genomic medicine seems far from relevant to clinical practice and therefore their engagement with educational activities is likely to be low? What might be the important, clinically-relevant topics to address in educational activities that medical specialists identify as being critical to their adoption of genomic investigations? Also of importance is understanding non-genetic medical specialists' preferences and expectations for their future practice of genomic medicine, as this will also provide insight into their needs for continuing education which can be specific to their clinical role.

Existing, published surveys address some of these research questions. Some focus on genetic concepts (e.g., taking family history) and tests (Jenkins et al., 2010; Calzone et al., 2012) and others are specific to local context (i.e. specialty/discipline or health service) (Bonter et al., 2011; Haga et al., 2012; Stanek et al., 2012; Marzuillo et al., 2013; Helman et al., 2016; Chow-White et al., 2017; Groisman et al., 2017; Johnson et al., 2017; McCauley et al., 2017). For example, Chow-White et al. (2017) surveyed oncologists' attitudes towards genomics and McCauley et al. (2017) focused only on physician training in genomics. These are not suitable without adaptation to be deployed across a diverse range of disciplines or services. There are no published surveys that cover the breadth of our research questions in the context of genomics.

We therefore aimed to develop an evidence-based survey that could be disseminated to a national sample of non-genetic medical specialists across diverse sub-specialties in Australia to ascertain their rationale for their practice of genomic medicine with a focus on their training needs. The purpose of this article is to describe in detail the methodology for developing this robust survey. Survey development was informed by literature (Chen and Kim, 2014; Gray et al., 2014; Chow-White et al., 2017; Carroll et al., 2019; Nisselle et al., 2019a; Stark et al., 2019b), theory and qualitative data, has had input from experts for content validity and was reviewed by non-genetic medical specialists representing target respondents for usability and functionality.

## Materials and Methods

### Research Design

A mixed-methods exploratory sequential (survey development) design was used, involving an initial qualitative phase with key informant interviews (Creswell and Plano Clark, 2017). The qualitative findings then informed development of a context-specific quantitative survey for dissemination nationally to non-genetic medical specialists in Australia. Data collection using the survey across Australia has been completed and will be reported in a separate publication. This study had human research ethics approval (University of Melbourne, HREC: 1646785). As per the approved research protocol, interview participants gave verbal consent for interviews to be audio recorded, transcribed and for de-identified quotes to be used in publications or reports arising from the research.

### Qualitative Phase: Key Informant Interviews

#### Sample

Two sample groups were approached for key informant interviews: those who provide continuing education in genomic medicine to medical specialists (‘education providers') and medical specialists as the target learner group.

#### Education Providers

Individuals and organizations providing genomic education were identified through a desktop audit mapping relevant genomic educational activities or resources in Australia (McClaren et al., 2018). The desktop audit identified 59 distinct genomic educational interventions (37 substantive ongoing programs or resources; 20 postgraduate course or single subjects; two massive open online courses). Where contact information was available on a website or advertisement, convenors of each identified educational intervention were invited to participate in a semi-structured interview. Those who responded were sent a plain language statement and consent form, and a phone or face-to-face interview was scheduled at their convenience. These interviews with education providers collected information about the participant, including formal qualifications and relevant experience leading to their becoming the convenor of the particular intervention. As well as information about the educational intervention, providers were invited to comment on future education needs in genomic medicine, and to discuss potential barriers and facilitators to meeting these needs.

#### Non-Genetic Medical Specialists

Details of recruitment and data collection with these participants is described elsewhere (McClaren et al., in press). Briefly, a national sample of medical specialists across diverse disciplines was recruited for semi-structured interviews.

Exclusion criteria were:

- Medical geneticists—we have conducted a separate study of genetic health professionals' workforce readiness (Nisselle et al., 2019a);
- General practitioners (GPs)—excluded due to the differences in practice between primary, secondary and tertiary care. We have undertaken a separate study with GPs to understand their current practice of genomic medicine, including their experience with direct-to-consumer/personal genomic testing (manuscript in preparation). The focus of the interviews and the approach to data analysis described in this article was to collect data to inform the design and development of future educational interventions for medical specialists to become skilled and competent to practice genomic medicine.

The interview guide for medical specialists explored current practice of genomic medicine, and interviewees' preferences for the future of genomic medicine relevant practice. Potential barriers and enablers to the integration of genomic medicine into practice may be areas for future educational interventions to address.

#### Data Collection

Interviews were conducted by authors BM or EC and Dr. Zoe Prichard, by telephone or face-to-face, digitally audio-recorded and transcribed *verbatim*.

#### Data Analysis

A thematic approach was initially taken to analyse transcripts. Authors BM and EC read and re-read the transcripts to identify similarities and differences in the conversations with participants, through constant comparison. The interview guide topics formed the basis of a deductive coding framework that was refined through discussion of emerging concepts which is inductive coding between authors BM, EC, AN, CG, SM (Corbin and Strauss, 2008). NVivo 12 was used to organise the data and manage coding (NVivo, 2018). All transcripts were systematically coded according to the developed coding framework.

### Selection and Refining of Survey Questions

**Figure 1:**
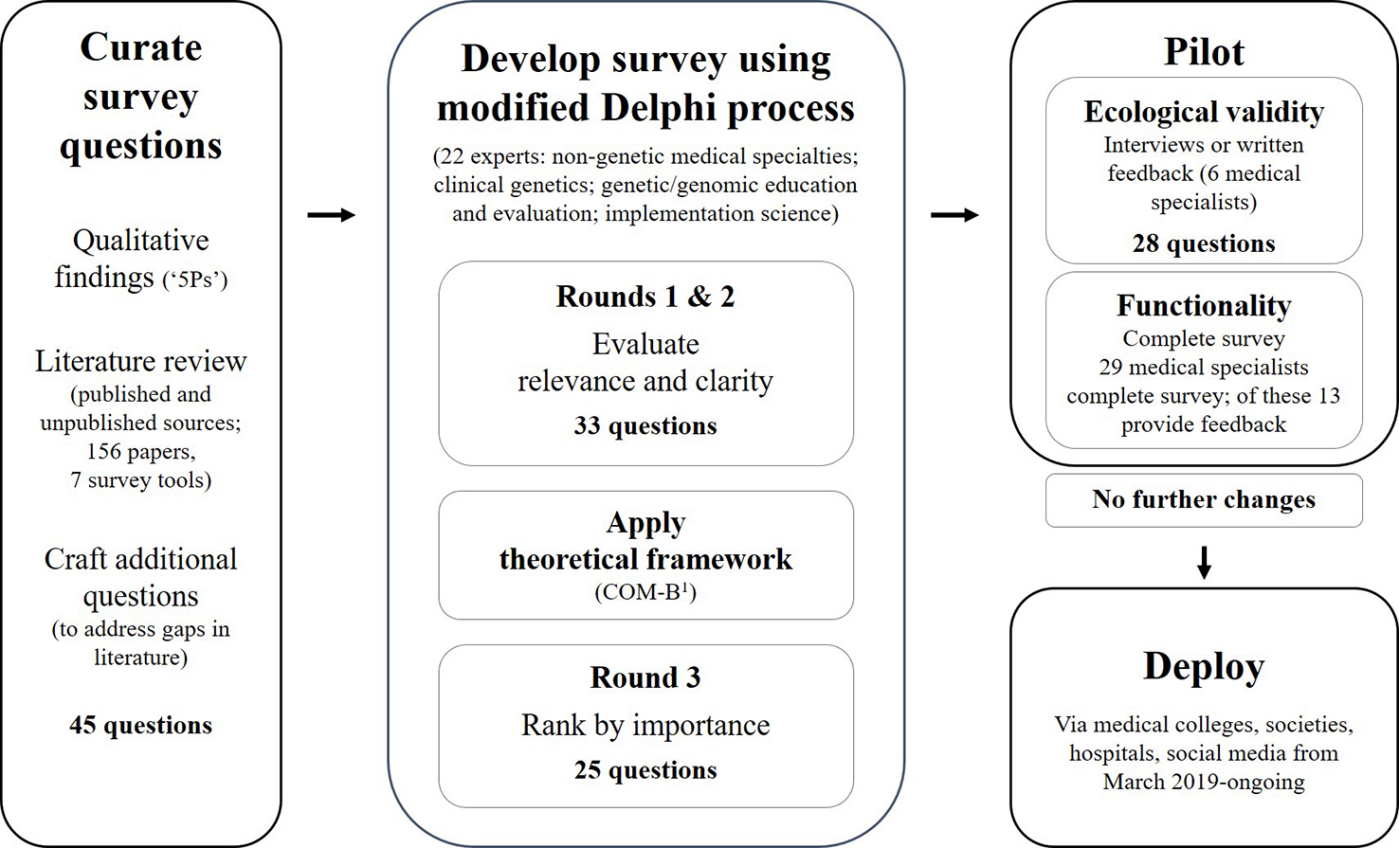
The survey development process: curate survey questions using qualitative findings, review of literature and craft additional questions; review questions using a modified reactive Delphi approach; pilot for usability and functionality; and, deploy the final survey (Michie et al., 2011)^1^.

The selection of survey questions and the process of refining these for use in the final survey (including a Delphi review by experts) is shown in **Figure 1**. The literature were searched for existing surveys that assessed genetic or genomic practice and/or education and training needs of medical specialists; these included peer-reviewed publications, government reports, student theses and conference abstracts, encompassing both published and unpublished surveys (Chen and Kim, 2014; Gray et al., 2014; Chow-White et al., 2017; Nisselle et al., 2019a; Carroll et al., 2019; Stark et al., 2019b). Relevant survey questions were collated and evaluated against concepts identified in the qualitative phase. If there were no, or few, questions in a category, new questions were developed with expert input from the Australian Genomics Workforce & Education working group (**Figure 1**), to generate a question bank for expert input through a Delphi process. A total of 25 questions were included and/or adapted from prior surveys and three new questions developed for the final survey. The breakdown of these were: n = 15, (Nisselle et al., 2019a); n = 5, (Stark et al., 2019b); n = 3, (Chow-White et al., 2017); n = 2, (Carroll et al., 2019); n = 1, (Gray et al., 2014); n = 1, (Chen and Kim, 2014).

In a traditional Delphi process, experts first generate a list of items and refine over subsequent rounds for relevance and clarity (McKenna, 1994). Given that the initial question bank was informed by qualitative findings and existing surveys, a modified, reactive process was used (McKenna, 1994). The question bank was refined using three rounds to: assess each question for relevance and clarity; modify or develop new questions if required; apply a theoretical framework; and, reduce length (Goodman, 1987; Streiner et al., 2015). Experts were selected purposefully representing four areas of expertise: non-genetic medical specialties; clinical genetics; genetic/genomic education and evaluation; and implementation science. The experts were recruited through research and professional networks of the Australian Genomics Workforce & Education working group, plus snowball sampling to ensure national representation. Each round was open for comment for two weeks, with two weeks between rounds for data analysis. The process and data from the Delphi rounds were managed using an online REDCap database hosted at the Murdoch Children's Research Institute (Harris et al., 2009). The online data collection tool simplified the feedback process for experts because REDCap can be used on computers and portable devices at different times, with save and return functions. Using an online approach was also more efficient for the analysis of responses as data could be collated and exported from REDCap.

#### Round 1: Review Relevance and Clarity

Experts reviewed questions in the initial question bank for clarity and relevance to the survey objective, and suggested edits if necessary. Questions were included in subsequent rounds if there was 80% expert consensus to keep the question. To ensure transparency of disparate opinions between professions, data were stratified and prioritized by areas of respondent expertise and re-presented for Round 2 review by the entire Delphi expert group. For example, if a question assessed use of genomics in medical practice and expert consensus was not reached, the data of non-genetic medical specialists were given priority over data provided by other expert groups for that question.

#### Round 2: Reject or Ratify Changes

**Figure 2:**
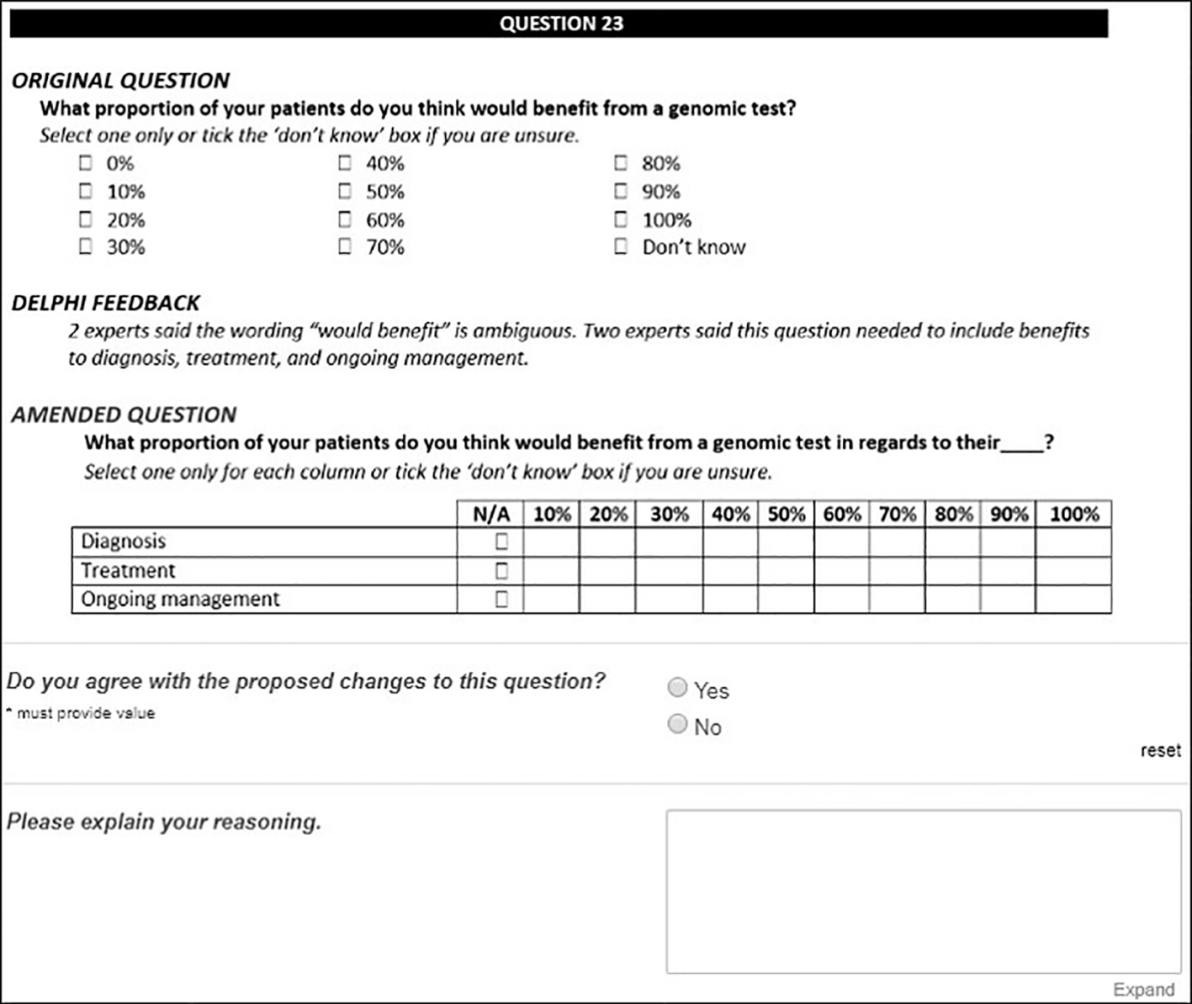
An example of Delphi expert tasks for Round 2 as shown in the REDCap online database.

Experts were shown aggregate Round 1 feedback and reviewed the original and the amended versions of questions. For this round, experts were asked to rate their agreement with any proposed changes and their perception of question relevance and clarity for inclusion in the final survey (**Figure 2**). Questions were included in the final survey if there was 80% expert consensus to keep. Questions were excluded if there was a unanimous decision to exclude. All remaining questions progressed to the next round for review.

#### Mapping Survey Questions to a Behavior Change Theoretical Framework

**Figure 3:**
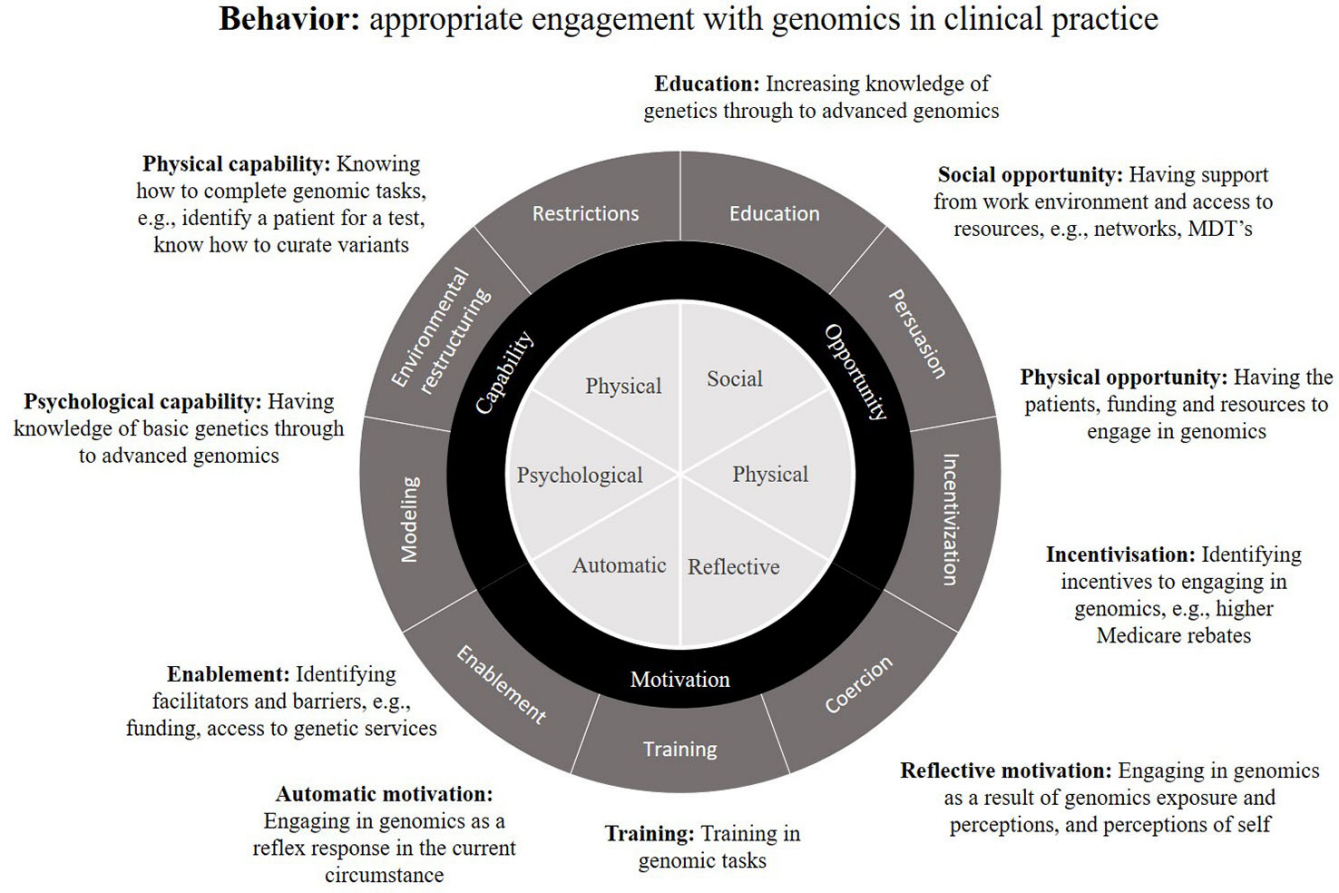
The Behaviour Change Wheel theoretical framework, encompassing capability, opportunity and motivation (COM-B), as applied to behavior defined as: appropriate engagement with genomics in clinical practice. Examples are given of potential behavior change interventions applicable to the practice of genomic medicine (adapted from Michie et al., 2011).

A rigorously developed survey grounded in theory facilitates translation of the survey across a range of settings. To ensure a sound theoretical underpinning for the survey, remaining survey questions were then mapped to Michie's theoretical framework for behavior change, the COM-B model (Michie et al., 2011). This framework was chosen because it is likely that the data collected with the developed survey will inform educational interventions to target behavior change for the practice of genomic medicine by non-genetic medical specialists. The model proposes that behavior change is a result of interaction between three factors relating to an individual—capability, opportunity and motivation (**Figure 3**). These factors are then embedded in the Behaviour Change Wheel tool, providing the translational step to bridge findings from data collection into clinical care and therefore appropriate as a theoretical framework for the survey.

For this study, we defined the target behavior as ‘appropriate engagement with genomics', given that use of genomic medicine varies by medical specialty and health service delivery context. Survey questions were mapped were mapped according to the following definitions.

##### Capability

The knowledge and skills required to engage in genomic medicine, ranging from knowledge of basic genetics through to advanced genomics and clinical skills required to refer/order testing, etc. Example survey questions include self-reported genomic knowledge and confidence, ordering genomic tests, and current genomic continuing education.

##### Opportunity

Environmental factors that support or hinder genomic medicine practice and cannot be resolved with education or training, e.g., work environment where genomic testing is implemented, peer support, access to resources. Example survey questions include access to genetic services, funding and education activities.

##### Motivation

An individual's perception of the benefits and limitations of genomic testing and how genomic information can guide patient care. Example survey questions include perceptions of self and genomics, and activities that increase awareness, e.g., exposure through research.

#### Round 3: Reduce Survey Length

**Figure 4:**
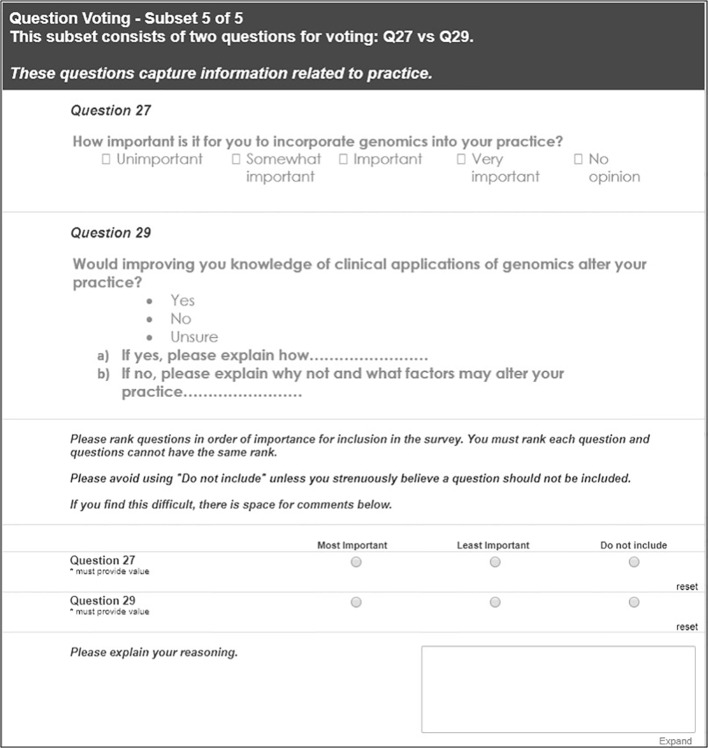
An example of Delphi expert tasks for Round 3 as shown in the REDCap online database.

Questions were then grouped by the initial qualitative phase concepts and/or COM-B domains to identify any redundancies. Delphi experts then ranked questions by importance within these groups to shorten the survey; an example of this process is shown in **Figure 4**.

### Piloting the Survey: Determining Face Validity and Functionality

First, face validity was confirmed. Members of the Delphi group nominated non-genetic medical specialists from their professional networks practicing in a range of settings, to review the final survey. Participants provided written or verbal feedback at the end of the survey on any aspects they found difficult to answer and/or could be improved; verbal feedback was collected in ‘talk-aloud' cognitive interviews (undertaken by EK) at a mutually convenient time (Czaja and Blair, 2005).

The final online survey in REDCap was then robustly tested for functionality. Non-genetic medical specialists who had been contacted to participate in the key informant interviews (qualitative phase) were re-contacted and invited to pilot the survey from 23 January to 15 February 2019. These specialists were asked to complete the survey in full to trial data capture systems in REDCap using a variety of devices and browsers; respondents could also provide optional feedback on their survey experience.

### Deploy the Survey: Data Collection

The online survey was open from February to September 2019 (manuscripts in preparation). A multipronged recruitment strategy aimed to reach as many medical specialists and trainees as possible across all career stages, Australian regions and specialties (excluding clinical genetics and general practice, as noted above). Additionally, this survey was not deployed to oncologists as the questions on genomic testing focus predominantly on germline testing; adapting the survey for oncology is the focus of further work. The survey was advertised via medical colleges, societies and hospital newsletters, member email distributions lists, internal communications and/or social media channels, and via Australian Genomics investigator networks and social media channels. All advertisements included a prompt to forward the survey link to relevant colleagues and respondents were also encouraged to share the survey among their Australian health professional networks.

## Results

### Qualitative Phase: Key Informant Interviews

Contact details of 39 education providers were obtained from the identified educational activities and 32 convenors responded and were interviewed (Janinski et al., 2018). Interviewee qualifications, which could be multiple, included nine clinical (genetic counseling, medical specialty, nursing or allied health), four pathology, and 24 doctorates (PhD) in science, social science or bioinformatics. Four interviewees held a tertiary qualification in education. The providers developed and/or delivered a wide range of educational interventions. These were (from most common to least common): continuing professional development (CPD) activities, formal education (e.g., university courses) or online courses/resources. Attendees or users of these interventions ranged from undergraduate students (e.g., medical, science, bioinformatics) to non-genetic health professionals, medical scientists and genetic specialists.

As described elsewhere, 240 medical specialists were contacted for interview and, of these, 86 were interviewed (McClaren et al., in press). The medical specialties included: anesthesiology, cardiology, dermatology, endocrinology, fetal medicine, general medicine, hematology, immunology, infectious disease, intensive care, nephrology, neurology, neuropsychiatry, obstetrics & gynaecology, oncology, general pediatrics, pathology, and rheumatology.

Interviewees were classified using their descriptions of current practice of genomic medicine and perceptions of their level of genomics experience, as: ‘novice' (no (or rare) use of genomics in clinical practice and/or; no involvement in genomics research and/or; ambivalence towards continuing education in genomic medicine), ‘interested' (infrequent use of genomics in clinical practice and/or; some (or rare) involvement in genomics research and/or; interest in, but perhaps not attendance at, continuing education in genomics) or ‘experienced' (current use of genomics in clinical practice and/or; active involvement in genomics research (molecular or clinical) and/or; participation in continuing education in genomics). These classifications, as well as the medical specialty, are shown as participant descriptors throughout to give context for illustrative quotes. The spread of self-reported genomic experience was: novice, n = 29 (34%); interested, n = 34 (39%); and experienced, n = 23, (27%).

Analysis of the transcripts from these 118 interviews with education providers and medical specialists resulted in five emergent (‘5P') concepts, which also formed the framework for survey development:

1. **p**ersonal characteristics (e.g. medical specialty, years of practice);
2. current **p**ractice of genomics in clinical and research settings;
3. perception of how **p**roximal genomic medicine is to practice;
4. perception of **p**reparedness (knowledge and confidence); and,
5. **p**references for
  - future roles and models of care in genomic medicine; and
  - continuing education in genomic medicine.

Interview quotes are used in the sections below to illustrate the concepts. Some quotes have been truncated for readability without changing the meaning, indicated by “…”.

#### The ‘5Ps’: Key Concepts Relevant to the Integration of Genomic Medicine Into Clinical Practice

##### Personal Characteristics

Personal characteristics of the medical specialist participants influenced their description of their readiness for genomic medicine. These included: medical specialty, types of patients seen (adult/children; public/private settings) and years of clinical practice. Participants also described how teaching roles contributed to their understanding of genomics.

*I used to teach undergraduate genetics for years … I would not, by any stretch of the imagination, attest to be an expert in these things, but I probably have a better background than most of my contemporary colleagues working here, just because of what I had done along the way. [MS36, mid-career, interested, nephrologist]*

##### Current Practice of Genomics in Clinical and Research Settings

Clinical practice of genomic medicine ranged from limited to regular use amongst the medical specialist participants interviewed. Participants from specialties including cardiology, hematology, neurology, intensive care and oncology described how genomic medicine has high relevance to patient care.

*My clinical practice is predominantly epilepsy and therefore, everything epilepsy has some genetic relationship, be it primarily genetic or the structural-vein abnormalities that also have genetic bases. So I guess a lot of my consultations do involve … some discussion at some point about the genetic contributions to the aetiology, be it complex genetics or single genes or somatic … it's a big part of my practice, not always possible to test for genes, but even just discussions with families around our understanding of the genetic contribution. [MS27, senior, novice, neurologist]*

*If a patient comes along, for example with melanoma, there's a handful of specific mutations that are known drivers of that disease. And we perform genomic studies to see whether those mutations are present. If they are present, then those mutations indicate specific therapies. If they're not present then we don't give the patient those therapies. [MS49, senior, interested, oncologist]*

Other participants described their perception of a lesser relevance of genomic technology approaches to their care of patients in fields including immunology and nephrology.

*We’ve been slow to move into this field in that, historically, a lot of the genetic disorders that we receive have come through to us from the pediatricians, often with a diagnosis, or there hasn't been (a need for) a genetic diagnosis, because (the patient) would either have a clinical diagnosis and the management would be just a pragmatic one of, trying to fix whatever was wrong or trying to manage the complications of their kidney impairment and therefore actually having the genetic diagnosis wasn't changing our practice. [MS36, mid-career, interested, nephrologist]*

It was evident from the interviews that some participants had gained knowledge and skills about genomics through avenues other than their clinical role. In particular, participants gained experience through their laboratory or clinical research involvement.

*I’ve been a (funding body details) researcher for the past 14 or 15 years. I have a background in genetic analysis … and we associate polymorphisms with risk of skin cancer, including melanoma. So I have a fairly good understanding of genetics and risk association, but not in a clinical setting as such. [MS22, senior, interested, dermatologist]*

The most commonly described approach to current practice of genomic medicine was to refer patients to a specialist Genetics service which is consistent with their clinical guidelines to promote appropriate requests for genomic testing.

*My two main areas of interest are gastrointestinal malignancies and breast cancer and certainly I'm sort of well aware of the guidelines for familial cancer screening and often refer a number of patients to the familial cancer centres. [MS35, early, novice, oncologist]*

*We have a very strong link with the Clinical Genetics unit….My colleagues and I have found it very helpful to refer first rather than to order the tests straight up. [MS64, mid-career, interested, clinical immunologist]*

*I look after a lot of children with genetic issues, or children with undiagnosed syndromes or medical conditions that are unexplained….I would, order the microarray and (single gene test for) fragile X. And then if I am concerned and haven't found results from there, from that then I send (refer) to Genetics. [MS54, early, novice, pediatrician]*

Fewer participants described specialist-led clinics that had a particular emphasis on the inherited or genetic aspects of patient care.

*In the clinic I have a dedicated interest in hereditary endocrine conditions so my clinic is skewed towards genetic conditions. [MS03, senior, interested, endocrinologist]*

##### Perception of How Proximal Genomic Medicine is to Practice

Participants were asked to describe their perception of how near in the future genomic medicine was likely to be part of their clinical practice. For some medical specialists, genomic medicine was not something they anticipated in their practice for quite some time.

*From a clinical day-to-day practice perspective, it doesn't really have much role at present. [MS24, senior, experienced, hematologist]*

*Within our clinical practice we would use genomics mainly in the context of endocrine tumours but there is no, currently, provision of testing for genetic mutations in endocrine tumours where we are in (name of state)….We are just starting to use panel sequencing for bone fragility but this is despite the fact that we showed in our research that you could do it just as efficiently with whole exome sequencing, which costs a whole lot less. [MS05, senior, experienced, endocrinologist]*

The varied proximity of genomic medicine to different specialties was echoed in the perspective of education providers.

*In the renal space for example, genomics and genetic testing hasn't hit them in a big way, you know whereas a cardiologist is much more aware of genetic testing and the benefits and limitations and all of that in their field. [EDU010, convenor of ongoing program/resource]*

There was, however, a sense that genomic medicine would become part of clinical practice, or was already being established.

*It is going to pervade everything we do……particularly as it becomes more and more mainstream and more equipment becomes cheaper and cheaper it is going to be more diagnostic, so personalized medicine and diagnostics in hospitals. [EDU018, convenor of university course/subject]*

*In the last 2 or 3 years it's come up more……I think it's a field in its infancy, it's growing and it's going to find more applications. And when we know more about it, we're likely to use more, and I think it's certainly got a role and it's only going to expand. [MS60, mid-career, interested, intensivist]*

##### Perception of Preparedness: (Competency and Confidence)

Participants described a perception that medical specialists were un- or under-prepared for future practice of genomic medicine.

*Many healthcare professionals not traditionally involved in genetic testing … their basic genetics 101…is not very strong, probably haven't used it for a very long time. The genetic potential that they learned 10–20 years ago was very much the classical style of genetics rather than what we know now from when a human genome project was finished … It's creating a lot of confusion … in practice as a healthcare professional. [EDU024, convenor of MOOC]*

Medical specialists identified that developing confidence to practice would be important for future integration of genomic medicine into clinical care.

*My confidence with the terms of the referral and feeling confident about what information I need to provide is much higher than my confidence in interpreting information … we absolutely rely on the expertise of the people writing the report in terms of variations of unknown significance … my confidence in terms of interpreting a VUS [variant of unknown significance] is very limited. [MS47, early, experienced, neuropsychiatrist]*

##### Preferences: Future Roles and Models of Care in Genomic Medicine

Medical specialists had a preference that if they are to practice genomic medicine in the future, then there should be a multidisciplinary team in place for optimal patient care, in particular where the testing may have a predictive application.

*Our genetic tests are ordered in conjunction with a multidisciplinary clinic that I run with my clinical genetic colleagues, and genetic counseling is conducted as part of that clinic. It is especially true for cancer syndromes. The Clinical Genetics department (here) has instituted what I think should be the gold standard process of gatekeeping where they will allow specialists from outside Clinical Genetics to order a genetics test on the proviso that adequate genetic counseling has been provided to the individual with the syndrome and that any positive test will then trigger Clinical Genetics review of predictive testing of family members … There is simply not enough space in Clinical Genetics to work but the clinical geneticists at our hospital are confident enough in the endocrinologists to be able to order a test for someone with a clinical syndrome, a phenotype where the risks of genetic testing are low because if you already have the phenotype you can't further damage the person by a molecular diagnosis. It is testing the asymptomatic individual where the risks have to be very carefully articulated. [MS03, senior, interested, endocrinologist]*

*They (Clinical Genetics department) certainly assist in making sure we order the right tests from the right lab … I think that's quite tricky. And they also have counselors … which means that I feel more confident that my patients getting the right information … And I certainly intend to keep using them because I think the patient gets better care. [MS64, mid-career, interested, clinical immunologist]*

*(We need to) encourage medical specialists to take this on and take it and work in partnership with each Clinical Genetic service … have some realization of the different types of tests and, “Gosh, I need to talk to someone about this'. Where a panel is appropriate here, and a single exome there, and a whole genome for that. [EDU019, senior, convenor of ongoing program/resource]*

The emergence of genomics experts within specialties was proposed as a future model of practice in which a specialist gains specific expertise in genomics as relevant to their patients.

*In my opinion the best model of care in terms of integrating genetics into clinical practice is to have specialist-led Genetics clinics where people…. Just like the specialists in cardiology who do angiograms and stick tubes in groins, I don't do that, that's not my specialist area. I'm a cardiologist but I don't do that. There should be a specialist for cardiology genetics, a specialist for neurology genetics, that sort of thing. That model of a specialist-led Genetics clinic is the best model because the phenotype is so important, you have to get the phenotype right before you can interpret any genetics information. [MS06, senior, experienced, cardiologist]*

*There just aren't enough geneticists or genetics counselors to deal with all the data that's going to be coming in in the next few years. I'm a strong believer that each discipline needs to understand the genetics of its disorders going forward. [MS23, senior, experienced, neurologist]*

This was further emphasised by medical specialists wanting to manage genetic investigations for common or ‘minor' conditions.

*I think that I am quite capable of speaking to people about testing their family without involving genetic counselors. I actually don't need to have them involved for those minor genetic disorders. So it really depends on, I think, the clinical significance of the genetic disorder. [MS24, senior, experienced, hematologist]*

##### Preferences: For Continuing Education in Genomic Medicine

The most valued approach described by educators and medical specialists was for learning through continuing education when there was the opportunity to gain ‘hands-on experience’.

*I think that you really need hands-on experience, you have to have a mixture of didactic lectures, case examples, and hands-on experience, people rotating through workshops … You don't have to curate, but getting in there, and doing a couple helps you understand the process, helps you understand the complete process … If people understand the process, then I think they get much more out of the MDT (multidisciplinary team) meetings. [EDU007, convenor of ongoing program/resource]*

*Speaking as a clinician it would be important to me that it [education] had a practical focus … It could still be lecture-based or small group-based … But you know, clinically, practical-focused. [MS43, senior, interested, nephrologist]*

There was also a strong preference from participants that continuing education is delivered in a clinically relevant way, although they recognized that this was challenging as different medical specialties, and individual specialists, would have different perceptions of what is clinically relevant.

*I talk to a lot of people in my role and this goes all the way from genetic counselors to clinical geneticists, medical specialists … the information they require I find really differs depending on what field they're in … the different fields and different specialists are at very different stages and requirements. [EDU010, convenor of ongoing program/resource]*

*Everyone would love to have time to get educated but the reality is attendance to that sort of activity often comes second, particularly when you've got busy clinics and patients coming through. But if you have a patient who is really challenging you, and you have the opportunity to improve the management of that patient if you go along to this tumor board (meeting), then you all of a sudden have another reason why you should attend when the forum is integrated with basically patient care. [MS49, senior, interested, oncologist]*

*As long as it's clinical, you know, we all get basic genetics at university but it's sort of how it's applicable to clinical practice that matters most for clinicians. [MS51, mid-career, interested, neuropsychiatrist]*

#### Overlap and Intersection of the 5P Concepts

The 5P concepts that formed the framework for the survey development can be considered separately as shown in the section above, but they do intersect and overlap. For example, as shown in **Box 1**, a medical specialist perceives genomic medicine to be very proximal to their practice because they currently include genomic investigations in their usual care. In doing so, they have experience in requesting genomic investigations, receiving reports and interpreting results for their patients. This experience may contribute to their perception of being prepared. Medical specialists in these contexts may have different preferences for continuing education than other specialists who do not currently request genomic investigations and/or do not anticipate doing so in the near future (genomic medicine is distal to practice).

Box 1 **An example of overlapping 5P concepts, illustrated with quotes from an experienced, mid-career clinical immunologist who sees adult patients [MS65].**

Genomics **proximal** to their **practice** (special interest in primary immunodeficiency). **Proximity** motivated them to upskill (to become **prepared**)

*One of the main areas, I think, with primary immunodeficiencies is clearly the genomics and that side of the field. So I started to get interested in it from there*.

*Immunologists are quite diverse so there are some people who don’t do a lot of immunodeficiency or autoinflammatory and deal mostly with allergies, and that’s their interests. But certainly there’s a lot of interest around where I am*.

Having experience with genomic testing in their **practice** has contributed to perceived **preparedness** (competence and confidence)

*I probably do have a reasonable understanding of the technology, and as I said, I do have some exposure to the technology through my other work (in pathology)...The technology itself is something that takes a bit to get your head around. And I obviously see the type of immunodeficiency patients, I’ve got some clinical involvement, so I think I am managing to keep up with it (genomics)*.

**Preference** for future model of care is influenced by their **practice**; experience has suggested a multidisciplinary model of care works best and they want this to continue because it provides opportunity to learn from peers (**preference** for education).

*It’s a complicated thing immunodeficiency. You need someone with expertise in that as well as someone with genetics expertise...I think there’ll be more collaboration (going forward)*.

*It’s always helpful to have collaboration with the, sort of genetic scientists, clinical geneticists and the involvement of the genetic counselors in the process. All of those things definitely help (to navigate genomics), it’s kind of a hard to do as a single practitioner*.

### Quantitative Phase: Survey Development

#### The Delphi Review

Twenty-six experts were contacted to participate in the Delphi review of the initial survey question bank. Of those invited, 22 agreed to participate (**Figure 1**). The final Delphi expert group comprised six medical specialists, nine genetics specialists, six genomic educators, and one implementation scientist from across Australia. Of the 22 experts recruited, 17 completed all three rounds of the modified Delphi process. The numbers of questions at each round are shown in **Table 1**. See **Table 2** for an example of a question Delphi feedback and modifications throughout the rounds.

**Table 1:** Numbers of survey questions throughout the Delphi rounds and after piloting.

Delphi Round	Personal	Practice	Proximity	Preparedness	Preferences	C	O	M	B	Total
Round 1	11	8	16	15	9	15	13	16	5	45
Round 2	10	13	11	14	7	12	10	12	5	33
Round 3	9	13	9	13	6	10	13	8	6	25
**Final survey**	12	8	11	14	7	12	12	8	7	28

Questions are mapped to more than one concept or domain; C, capability; O, opportunity; M, motivation; B, behavior (Michie et al., 2011).

**Table 2:** An example of question evolution using a modified Delphi process and mapping questions to the COM-B framework.

**Round 1 (relevance and clarity)**	
Original question	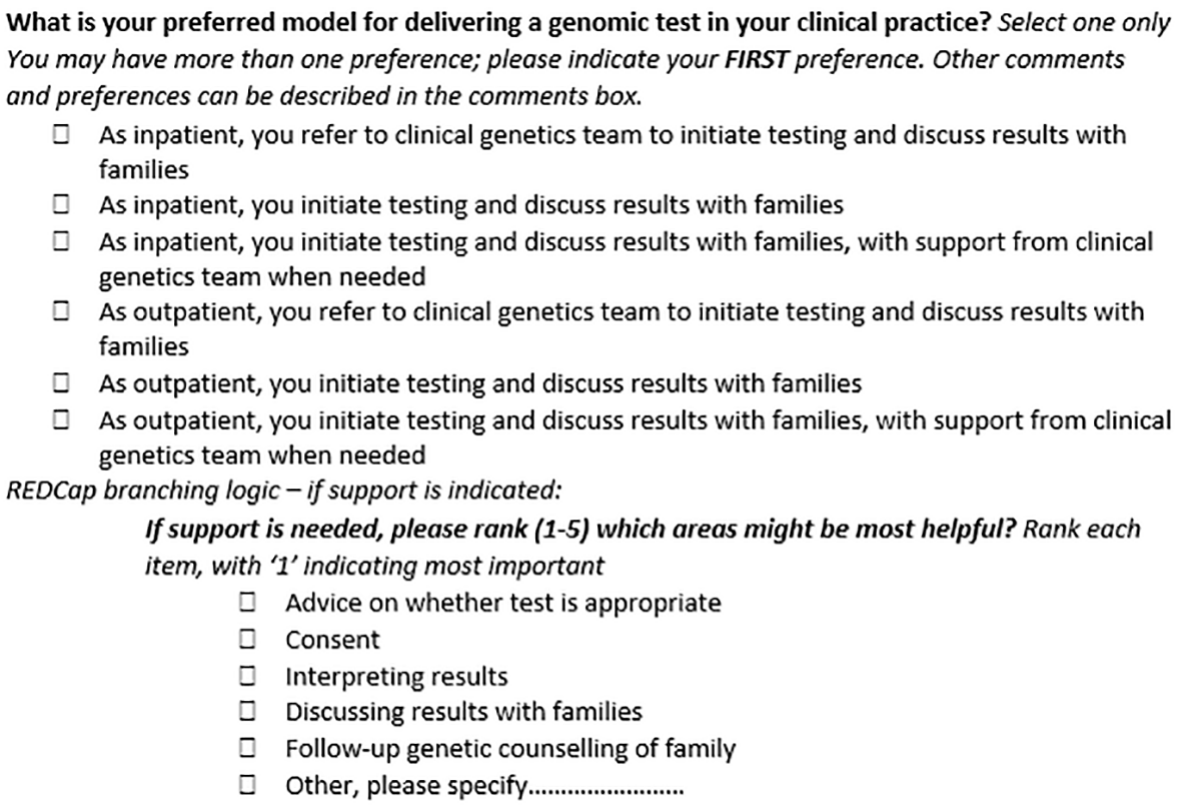
Instructions	Do you think this question is relevant to the aims of this sub-section? (Yes or No) Do you think this question is clear? (Yes or No) Are there modifications you would make to this question?….
Rating	All Delphi experts (100%) said this question was relevant and most (80%) said it was clear When stratified, only 40% of medical specialists rated as clear
Comments	Medical specialist: *“…preferred model surely depends on whether the patient is an inpatient or outpatient…”* Genetic specialist: *“omit inpatient/outpatient as other specialties would see both or purely outpatients”*
Outcome	Medical specialist responses prioritized and changes made in line with their comments
**Round 2 (ratify or reject changes)**	
Updated question for Round 2 review	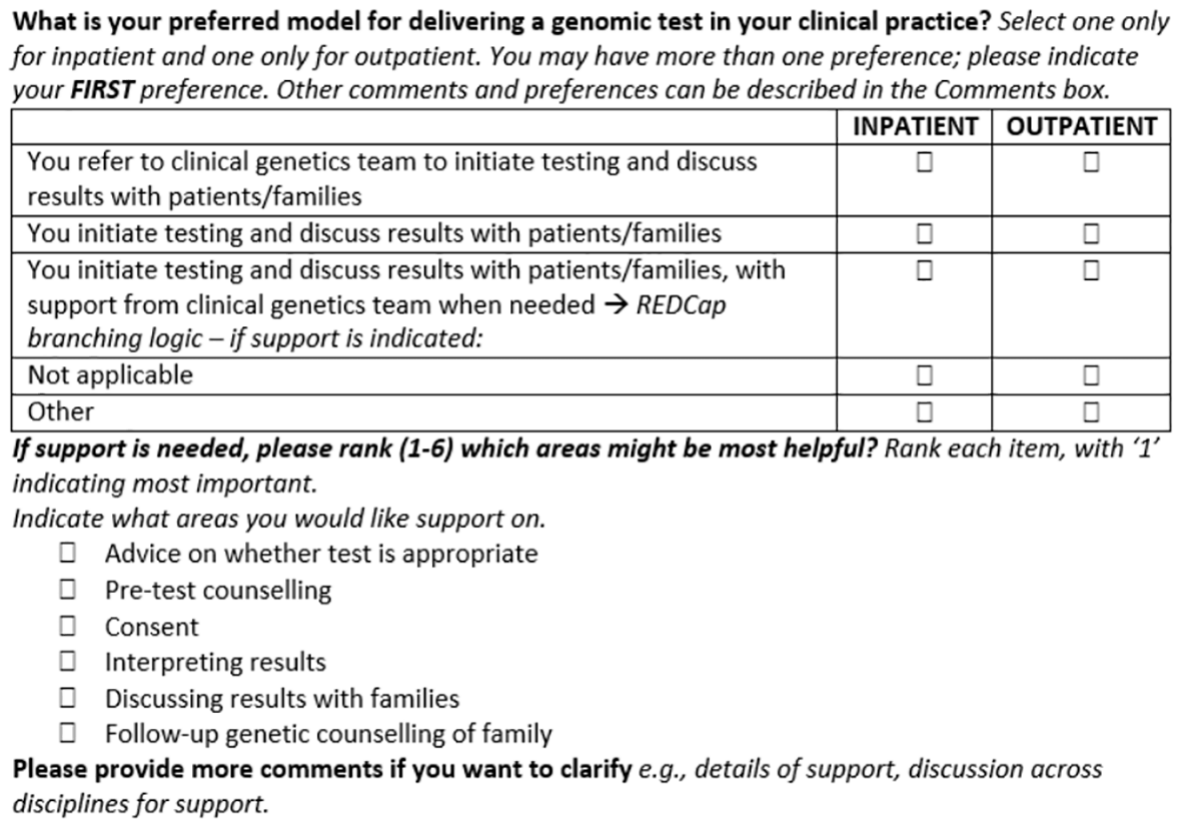
Instructions	Do you agree with the proposed changes to this question? (Yes or No) Do you think the amended question is clear? (Yes or No) Please explain your reasoning….
Rating	All experts (100%) agreed with the change to the question and most (95%) thought the amended question was clear
Comments	Medical specialist: *“The ranking system is helpful as is separating in- and outpatient. Also would change order - You initiate, you initiate and get support, you refer, N/A, other”*
Outcome	Question accepted as final after minor changes to wording.
**Mapping to COM-B model**	This question mapped to the domain ‘Behavior' as it assesses preferred level and method of engagement in the behavior
**Round 3 (reducing survey length)**	
Final question for Round 3 ranking	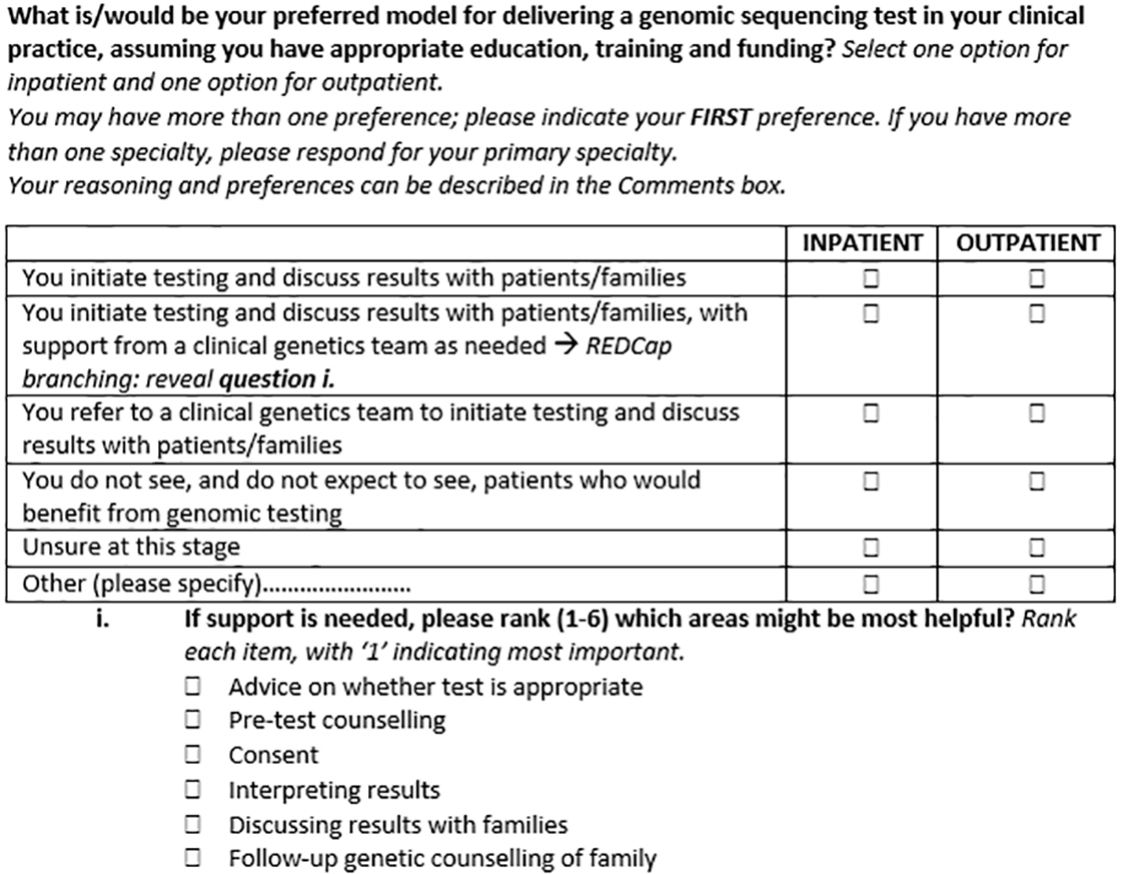
Instructions	This question was included in a subset of four questions for ranking to determine inclusion in final survey. All four related to practice (5P) and behavior (COM-B)
Rating	Question ranked as second most important in the subset to include in the final survey
Comments	(none)
Outcome	Following discussion with Australian Genomics Workforce & Education working group, this question was retained

C, capability; O, opportunity; M, motivation; B, behavior (Michie et al., 2011).

#### Round 1: Review Relevance and Clarity

All experts completed Round 1, which included 45 questions for review. The experts reached agreement (consensus) on relevance and clarity for five questions, which were retained to be included in the final survey. For three questions where consensus was less than 80% and qualitative feedback unanimously excluded the questions, these were removed. For the remaining 37 where there was less than 80% agreement but qualitative feedback was varied, written feedback from prioritized perspectives was used to amend questions (see **Table 2** for an example). Questions that addressed similar concepts were combined and the Delphi experts suggested four new questions, which brought the total number of questions requiring further review to 27.

#### Round 2: Reject or Ratify Changes

Eighteen experts completed Round 2. Of the 27 original questions presented for review, 25 were included, two were excluded. The three extra questions suggested from Round 1 were also reviewed and agreement reached to include these. In total, after Round 2, there were 33 questions remaining (five questions had already reached consensus in Round 1).

#### Map Questions to the COM-B Model

The results of mapping of questions to the COM-B theoretical framework (**Figure 3**), are shown in **Table 3**.

**Table 3:** Final survey questions mapped (shown with an X) to concepts from the initial qualitative findings and the domains of capability, opportunity, motivation and behaviour of the behaviour change wheel theoretical framework (Michie et al., 2011).

No.	Survey questions	Personal	Practice	Proximity	Preparedness	Preferences		C	O	M	B
						Future practice	Education				
**1**	What is your gender?^a^	✖									
**2**	What is your age bracket?^a^	✖									
**3**	Where are you located?^a^	✖									
**4**	Do you see patients in your practice?^a^	✖									
**5**	What is your current level of specialty certification?^a^	✖									
**6**	In what year did you complete your medical degree (MBBS/MD)?^a^	✖									
**7**	What medical specialty are you qualified for, accredited in or studying towards?^a^	✖									
**8**	Which categories of patients do you see?^a^	✖									
**9**	Who is your main employer?^a^	✖									
**10**	In the last 12 months, what was your main work location?^a^	✖									
**11**	Do clinical guidelines exist for genomic testing in your specialty?^b^		✖	✖	✖			✖	✖		
**12**	Have you been involved in any genomic research projects in the last 5 years?^a^	✖	✖	✖	✖				✖	✖	
**13**	Have you contacted your clinical genetics team or service in the last 12 months?^c^		✖		✖			✖	✖	✖	✖
**14**	Did you order chromosomal microarray (microarray) tests in the last 12 months as part of your clinical or research role?^d^		✖	✖	✖			✖	✖	✖	✖
**15**	Did you order gene panel tests in the last 12 months as part of your clinical or research role?^d^		✖	✖	✖			✖	✖	✖	✖
**16**	Did you order whole exome or whole genome sequencing tests in the last 12 months as part of your clinical or research role?^d^		✖	✖	✖			✖	✖	✖	✖
**17**	Below is a list of some of the steps involved in genomic sequencing testing from pre-test to post-test. Please indicate which steps you currently perform and which ones you expect to perform in the future if you had adequate education, training and support.^a^		✖	✖				✖	✖		✖
**18**	What is/would be your preferred model for delivering a genomic sequencing test in your clinical practice, assuming you have appropriate education, training and funding?^d^ [Options for Inpatient vs Outpatient]			✖	✖	✖					✖
**19**	Below is a list of ways genomic sequencing tests and other genomic tests can be initiated and discussed with patients. Please indicate which currently occur in your practice and/or you believe will occur more frequently in the next five years.^a,e^		✖	✖					✖		✖
**20**	Do you think genomics will impact your practice in the next 2 years?^b^			✖							
**21**	Do you feel prepared to use genomic sequencing testing in your practice?^b^				✖			✖		✖	
**22**	How confident are you in your: knowledge about genomics; ability to elicit information in a family or medical history; ability to explain concepts; ability to make decisions based on genomic information? What would help improve your confidence?^f^				✖		✖	✖	✖	✖	
**23**	Would improving your knowledge of genomic medicine alter your practice?^e^				✖	✖			✖	✖	
**24**	Have you ATTENDED any professional development education or training around genomics in the *past year*, such as lectures, seminars or workshops, either in person or online?^a^			✖	✖		✖	✖			
**25**	Have you PROVIDED any professional development education or training around genomics in the *past year*, such as lectures, seminars or workshops, either in person or online?^a,c^	✖		✖	✖			✖			
**26**	Who should be responsible for updating medical specialists about genomics?^e^						✖				
**27**	Below is a list of activities that can be used to keep up to date with, or learn new skills in, genomic medicine. Please indicate which activities you currently use and/or would prefer to use to keep up to date with, or learn new skills in, genomic medicine.^d^				✖		✖	✖	✖		
**28**	Below is a list of education topics in genomic medicine. Please indicate which topics you have learnt about and which you want to in the future.^g^				✖		✖	✖	✖		

Existing surveys were reviewed and relevant questions were selected (and modified in the Delphi rounds) for inclusion in the developed survey. Original sources were: ^a^(Nisselle et al., 2019a); ^b^Crafted by Australian Genomics Program 4 Working Group; ^c^GECKO survey (Carroll et al., 2019); ^d^(Stark et al., 2019b); ^e^(Chow-White et al., 2017); ^f^(Gray et al., 2014); ^g^(Chen and Kim, 2014); C, capability; O, opportunity; M, motivation; B, behaviour (Michie et al., 2011).

#### Round 3: Reduce Survey Length

In Round 3, the survey was reviewed for overall length to be mindful of the time it would take for respondents to complete. All demographic items and three questions assessing involvement in genomic research, awareness of clinical guidelines and confidence in genomic knowledge, were deemed essential for inclusion by the Australian Genomics Workforce & Education working group and so were not reviewed by the Delphi group for potential exclusion from the final survey. The remaining questions were organised into groups based on the 5P concepts and/or aspects of the COM-B domains (**Table 3**).

Seventeen Delphi experts ranked the questions within each subset by preference of inclusion in the final survey. Where consensus was reached, the questions considered most important were included in the survey (**Table 1**). Where there was a lack of consensus, the Australian Genomics Workforce & Education working group reviewed rankings and feedback to decide which questions to include in the final survey.

### Piloting the Survey

#### Face Validity

To obtain feedback from non-genetic medical specialists (the target population for the final survey), the Delphi group nominated colleagues who were then invited to provide insights on face validity. Five participated in the talk-aloud ‘cognitive' interviews and one completed written feedback (Czaja and Blair, 2005). These medical specialists were from three Australian states and five specialties. Feedback suggested alterations to question response options (e.g., lists of specialties) and gathering more in-depth information about contact with genetic services and level of engagement with education and training. Questions to address the last two suggestions were sourced from the GEC-KO Family Medicine Genetics Survey (Carroll et al., 2019).

Changes were also made to the survey at this stage to ensure data quality, e.g., adding a question to exclude respondents who did not practice clinically.

#### Functionality

The survey was sent *via* email to 240 addresses of those invited to initial key informant interviews, with 29 surveys completed online. Of these, 13 individuals provided additional detailed feedback on their use of the survey (ten written, three verbal). Feedback related to survey functionality in REDCap and clarifying ambiguity of questions or instructions. **Table 4** provides illustrations of feedback and subsequent amendments during functionality testing. For example, during an interview, a medical specialist commented that they did not know what a ‘rollover definition' was or how to use it despite this being explained in the introduction to the survey. These rollover definitions were crucial for appropriate and consistent interpretation of terminology in questions. To ensure definitions were read by all participants, the definitions were therefore also added underneath each question. Other minor changes were made to survey questions to improve participant understanding of questions before finalizing the survey for deployment.

**Table 4:** Examples of pilot survey feedback and amendments on ecological validity and functionality.

Question	Summarized feedback	Outcome
5) What is your current level of specialty certification? *Select all that apply, including options for dual trainees and sub-specialists, if applicable to your discipline*	‘Basic trainee' through to ‘Fellow' are concepts defined by medical colleges; sub-specialty is not	Removed ‘Fellowship sub-specialty' option
Basic trainee Advanced trainee Fellow Fellowship sub-specialty
7) What medical specialty are you qualified for, accredited in or studying towards?	Response options should be consistent with regional governing body	Changed list to that published by the Medical Board of Australia
17) Below is a list of some of the steps involved in genomic sequencing testing [rollover definition] from pre-test to post-test. Please indicate which steps you currently perform and which ones you expect to perform in the future if you had adequate education, training and support	Pediatricians may think of microarrays when asked about ‘genomic sequencing tests' so need to clearly specify this question asks only about whole exome or genome sequencing tests	Added instruction: *Note: this question does NOT relate to microarray or gene panel tests. We are only asking about whole exome/genome sequencing tests in the question*

The final survey is included as ‘**Supplementary Data Sheet 1**—final survey' and, in sum, consisted of 28 questions, noting the source of questions or topic items from existing surveys (**Table 1** and **Table 3**).

## Discussion

This mixed-methods study describes the development of a survey designed to measure a wide range of non-genetic medical specialists' current practice of genomic medicine and their preferences for future practice and continuing education in genomic medicine. This instrument was used to survey a national sample of non-genetic medical specialists practicing in Australia (manuscript in preparation).

A strength of this survey is that it has an embedded theoretical framework and is informed by qualitative data collection. Using the concepts that emerged from the qualitative data as a framework for the survey has ensured that identification, selection and development of survey questions covers the breadth of topics related to current practice and needs for continuing education in genomic medicine. The qualitative analysis demonstrated the way in which these concepts can be considered individually but also importantly that there is overlap; sections of interview transcripts could be coded at more than one overarching concept (**Box 1**).

This overlap is also evident in the final survey questions (**Table 3**), which means that the patterns seen in the qualitative work, and how participants discuss issues of continuing education and future practice, have been maintained in the development of survey questions. The final survey is a flexible tool that can assess individual or multiple concepts simultaneously. The survey is therefore useful for a range of research questions. For example, using practice questions (single question or suite of questions) if data are required to demonstrate the current non-genetic medical workforces' use of genomic investigations; or only using the questions mapped to preparedness for non-genetic medical specialists in a particular setting.

Basing the survey development in an emergent qualitative framework and a theoretical framework means that the survey can inform the selection of, or identify the need for development of, educational interventions to support non-genetics medical specialists as they develop competence to practice genomic medicine. Data from this survey can determine if and how educational interventions need to be tailored to the needs of individual sub-specialties and even individuals within those groups, based on clinical need. The data collected using this survey will provide much needed detail for education providers about which specialties are likely to engage with and participate in education interventions; this will enable resourcing to be focused on creating specific elements. Resultant interventions should consider evaluating their learning objectives against core competencies such as those identified by National Coalition for Health Professional Education in Genetics^1^ (NCHPEG) which set out three domains from which a clinician can assess their practice and need for further education and training. However continuing education is not the only answer; a suite of interventions will be required for the effective integration of genomics into clinical practice (McClaren et al., in press; Paul et al., 2018; Crellin et al., 2019). This survey can contribute to identifying other key factors for which interventions may be targeted.

The modified, reactive Delphi process used in developing the survey allowed input from a geographically disparate, heterogeneous sample of experts. Individual feedback was collected in a structured manner using an online platform. Importantly, using a Delphi approach provided the opportunity for evaluation of group views during Round 2 to take the input beyond the individual and make use of the collective expertise. Further, in Round 3, questions were ranked to inform decision-making about the inclusion or exclusion of questions for the purpose of evaluating the length, and therefore the time required by potential respondents to complete the final survey.

The importance of including a pilot phase in survey development was highlighted in our study; ensuring functionality with future users is critical and assumptions must be tested, such as presuming users would understand how to access ‘rollover definitions' in the online survey platform. A possible limitation of the functionality testing approach we used is that we had a response rate of 12% in this final stage of survey development. This level of response is not uncommon in surveys with health professionals; Selkirk et al. (2013) report a similar response rate (13%) for email invitations of physicians to complete a survey about preparedness for genomics. Of the 29 users who tested functionality in our pilot, only 13 provided additional feedback. Ideally, testing functionality of a survey would be with larger number of the target population.

Comparing the qualitative data collected from education providers and non-genetics medical specialists proved challenging, as the data had different emphases: the education providers had few comments on current and future practice of genomic medicine, while the medical specialists had generally not participated in continuing education for genomic medicine so their preferences reflected hypothetical views rather than what has worked well for them in learning about the application of genomic technologies in their practice. We therefore prioritized the perspectives of different expert groups during the Delphi process for particular questions. This assumption was decided on as a way to resolve disparity in views about the survey questions but may have biased the results of the Delphi process. The Delphi experts were all very engaged with genomics, even across their perspective groups, and therefore may not represent fully the perspectives of the target group of all medical specialists.

Use of iterative review and applying theory in survey design has been previously described. Jenkins et al. (2010) used rounds of iterative review to develop a national survey of US physicians in genomics, based in Rodgers' Diffusion of Innovation theory. This theory was chosen by the authors because it is a useful framework to predict adoption of genomics and to guide the selection of genomic education interventions to support clinical practice. By contrast we selected the COM-B model to design a survey that would measure, at the level of the individual, concepts that influence their behavior in appropriate engagement with genomics in clinical practice. A rigorously developed survey grounded in theory facilitates translation of the survey across a range of settings, which can be used to draw comparisons across these settings. For example, Jenkins et al. (2010) then adapted their survey for nurses using a modified version of the methodology (Calzone et al., 2012). We are similarly adapting our survey for oncology and international settings using qualitative interviews with key informants to review the current survey questions and assess each for relevance and suitability, such as nuances of germline versus somatic testing and local health service contexts. The strength of our survey development process based in qualitative and theoretical frameworks means that changes to specific wording of questions can be made according to the setting in which the survey will be used but the questions can still be classified using the framework concepts, making comparisons between settings possible. Future users of the survey may review items for relevance to research questions and local contexts or needs, then amend or add items.

As has been previously described, education is not the only answer for the changes to behavior needed for non-genetic medical specialists to competently and confidently practice genomic medicine. Educational interventions, however, will be and should be used as part of such strategies (Nisselle et al., 2019b). For education to be part of any effective strategy, interventions need to be evidence-based, with focus and content informed by understanding of the needs of the target audience. These needs, as shown by our qualitative data, are related to the characteristics of the specialist, their current practice of genomic medicine, their perception of how proximal genomics is to their practice, how prepared they feel they are to practice and their preferences for future clinical practice and future continuing education. We have created a robust, data- and theory-informed survey which captures not only levels of experience, practice of genomics and preferences for education but also the challenges around engaging with education. Survey data will provide evidence for education providers to inform their interventions so that effective education can be available to contribute to establishing a medical workforce that is literate in genomics and more confident to competently practice genomic medicine.

## Data Availability Statement

The datasets generated for this study are available on request to the corresponding author.

## Ethics Statement

The studies involving human participants were reviewed and approved by University of Melbourne, HREC: 1646785. Written informed consent for participation was not required for this study in accordance with the national legislation and the institutional requirements.

## Author Contributions

BM, AN, SM and CG conceived the idea and design for the study, and BM and EC conducted the interviews and analysed the data. EK and AN led the survey development and the Delphi review. SM and CG, and the Working Group, provided advice throughout the study, and assisted with recruitment of experts for the Delphi group. BM drafted the manuscript and all authors revised drafts, approved the final version and agree to be accountable for all aspects of the work.

## Funding

This work was supported by the Victorian Government's Operational Infrastructure Support Program and a grant from the Australian National Health & Medical Research Council (GNT1113531); the contents are solely the responsibility of the individual authors and do not reflect the views of the NHMRC. EC was supported by the Helen R. Freeman Scholarship.

## Conflict of Interest

CG and SM are co-editors of the Research Topic where this manuscript is featured. All authors share their affiliations with CG and SM. CG and SM were not involved in the editorial or peer-review process; this was overseen by the third co-editor who is not affiliated with the authors' institutes.

The authors declare that the research was conducted in the absence of any commercial or financial relationships that could be construed as a potential conflict of interest.

## Footnotes

1
https://www.jax.org/education-and-learning/clinical-and-continuing-education/ccep-non-cancer-resources/core-competencies-for-health-care-professionals

## Supplementary Material

Supplementary Data Sheet 1 Australian Genomics 5Ps survey.docx.
