# Supplementary material for: Development of an Evidence-Based, Theory-Informed National Survey of Physician Preparedness for Genomic Medicine and Preferences for Genomics Continuing Education
Source: Front Genet. 2020 Mar 3;11:59. doi: 10.3389/fgene.2020.00059 (PMC7063665; doi:10.3389/fgene.2020.00059)
Supplement: Supplementary Data Sheet 1 — Australian Genomics 5Ps survey.docx. [file DataSheet_1.docx]

**Surveying medical specialists about their practice and training in genomics 2019**

**Information and consent to participate**

This survey is an activity of the Workforce & Education Program of the Australian Genomics Health Alliance (*Australian Genomics*). *Australian Genomics* is a NH&MRC-funded national network working towards the development of genomic medicine within Australia. The Workforce & Education Program aims to investigate current and future education and training needs of the workforce in genomic medicine.

This survey has three aims:

1. To assess experience with genomic testing in clinical practice
2. To assess medical specialist confidence, and preferences for models of practice for genomic testing
3. To examine current and future genomics education and training needs of medical specialists.

We will use these data to examine the impact of genomics on workforce training and planning. We’re particularly interested in medical specialists’ experiences and opinions around proximity, preparedness, and preferences for genomic medicine.

The target audience for this survey is **medical specialists who currently practice clinically in Australia.**

It doesn’t matter if you feel you don't know much about genomics, or don’t incorporate it into your practice at the moment; your opinions, views and experiences are valuable to us.

**Note:** there are separate studies for clinical geneticists, oncologists, pathologists, radiologists and general practitioners, who will be contacted directly.

***Please read about the survey in the information below. You can access the survey at the bottom of this page by clicking “Yes”. By clicking “Yes” you are providing consent to participate in this research study by completing the survey.***

This study has been approved by the Human Research Ethics Committee of the University of Melbourne (HREC 1646785.8).

**What will I be asked to do?**

Should you agree to participate, you will be asked to complete a survey. The survey will take about 15 minutes to complete. We appreciate it may be difficult to complete the survey in one sitting, so you can complete it over more than one session if needed. If so, you will need to make note of your individual Return Code to ensure your answers are saved and you continue where you left off.

**How will my confidentiality be protected?**

We will protect your anonymity and the confidentiality of your responses to the fullest possible extent, within the limits of the law. In addition, all responses are anonymous. If you choose to provide your name and contact details to participate in an interview at a later date and/or to receive a copy of the study findings, we will store these data separately from your survey responses

The information gathered in the survey will be reported in a collective way so that your individual responses cannot be identified. For example, as some demographic data could identify individual respondents in areas with few specialists, we will combine demographics data for some analyses.

Your responses will be stored on a password-protected computer at the Murdoch Children’s Research Institute in Melbourne, Australia for a minimum period of 7 years. At the end of this storage period all the data will be disposed of by deleting all computer files and backup files.

**What are the benefits of participating?**

You will have the knowledge that you have helped inform clinical practice around genomic medicine. By participating in this research study, you will also be helping to shape future education programs in genomics.

**What are the risks of participating?**

In the course of answering the questions in the survey, participants may identify gaps in either their knowledge or practice which may cause feelings of anxiety. If you do experience any of these feelings we can direct you to educational materials or you may contact one of the research team members, who will discuss your concerns with you: Dr Amy Nisselle (T 03 9936 6340) or Associate Professor Clara Gaff (T 03 9936 6504).

**Do I get a copy of the study findings?**

At the end of the study we will publish relevant findings in peer reviewed scientific journals as well as reports on the *Australian Genomics* website. At the end of the survey, you can choose to provide your details to receive a copy of the study results directly.

**Where can I get further information?**

Should you require any further information, or have any concerns about the content of this survey, please do not hesitate to contact:

- Dr Amy Nisselle, Specialist Project Officer, Australian Genomics Health Alliance, Murdoch Children’s Research Institute (T 03 9936 6340 or [amy.nisselle@mcri.edu.au](mailto:amy.nisselle@mcri.edu.au))
- Associate Professor Clara Gaff, Australian Genomics Health Alliance Program 4 Co-Lead, Murdoch Children’s Research Institute (T 03 9936 6504; E [clara.gaff@melbournegenomics.org.au](mailto:clara.gaff@melbournegenomics.org.au)).

Should you have any concerns about the conduct of the project, you are welcome to contact the Executive Officer, Human Research Ethics, The University of Melbourne (T 03 8344 2073; F 03 9347 6739).

**How do I agree to participate?**

By selecting the “**Yes**” checkbox below you agree to consent to completing the survey.

If you decide not to complete the survey now, we thank you for your interest in our study. You can always complete the survey at a later date.

| Please tick "**Yes**" if at least one of the following criteria apply to you.   - You gained, or are studying for, a medical specialist qualification in Australia and you see patients - You currently work as a medical specialist in Australia and you see patients. | - **YES**, and I consent to participate in this research by completing this survey. - **NO**, the inclusion criteria do not apply to me so I am ineligible to participate in this research. |
| --- | --- |

**Instructions**

**Thank you for taking part in this study.**

The survey is divided into three sections, with some sections being shorter than others:

1. Questions about you: basic demographics and medical training
2. Questions about your genomics practice (if any)
3. Questions about education and training

We’re particularly interested in medical specialists’ experiences and opinions around proximity, preparedness, and preferences for genomic medicine.

Some questions may appear repetitive but it is important that you complete as many questions as possible. We designed and piloted the survey to ensure we only ask questions that provide critical information to shape the future of genomic medicine in Australia. There is a blue progress bar at the top of each page and the survey automatically shows/hides questions based on your responses to minimise the time commitment.

**Navigation tips**

- Click on the Next and Previous buttons at the bottom of a page to save your answers and move to the next/previous page. **Don’t use the back button on your browser or keyboard as this will not save your answers.**
- If you are accessing the survey on a mobile device, the survey is best viewed in landscape (sideways) mode.
- You can complete the survey over more than one session. Click on the 'Return later' link to save your answers to date and come back later to answer. Make sure you write down the login code generated by REDCap, as you will need that code to access the survey you have already started. Use the same survey link, then click on the "Returning?" link in the top right hand corner and enter your code. (Note: you will need to type the code, not copy and paste). The survey will then open at the last page you completed. You can return as many times as you want, as long as you do not finalise the survey. Once you finalise, your unique link will not work.

Some terms are defined in the glossary and these appear with an asterisk (*) or dotted underline in the survey.

**Your responses will be strictly confidential.**

At the end of the survey you will have the following options:

1. provide your contact details to receive a copy of the results directly; and/or
2. provide your contact details to be contacted for telephone interview; or
3. complete the survey only and not provide any contact details.

If you choose to provide your name and contact, we will store these data separately from your survey responses.

For clarification of any questions please contact Dr Amy Nisselle, Specialist Project Officer, Australian Genomics Health Alliance, Murdoch Children’s Research Institute (T 03 9936 6340 or [amy.nisselle@mcri.edu.au](mailto:amy.nisselle@mcri.edu.au)).

# Glossary

Below is a list of terms defined for the purpose of this survey. Please read these definitions before completing the survey.

| **Term** | **Definition** |
| --- | --- |
| **Chromosomal microarray (microarray)** | Diagnostic genomic test to identify changes to copy number variants associated with disease. |
| **Gene panel** | Laboratory test to identify variants in several well characterised genes associated with a phenotype and clinical presentation. |
| **Genetic testing** | A genetic test investigates a single gene or a few genes, e.g., *CFTR, NF1, DMD, BRCA1*. |
| **Genomics** | Genomics is the study of single genes, their function and their interaction with all other genes in the genome and with the environment. |
| **Genomic sequencing testing** | A genomic test investigates many regions of the genome at once. For the purpose of this survey, genomic tests based on sequencing technologies are defined to include gene panels, whole exomes or whole genomes, but to exclude non-invasive prenatal testing using sequencing technologies. |
| **Genomic testing** | A genomic test investigates many genes at once. For the purpose of this survey, genomic tests not based on sequencing technologies are defined to include non-invasive prenatal tests and chromosomal microarrays. |
| **Queries about direct to consumer/personal genomic tests and/or online DNA testing** | This may be any query, e.g., general questions, testing for their condition, results from online DNA testing that patients bring to clinic and/or medical implications. |
| **Variant** | A change in a person’s DNA sequence. |
| **Variant classification** | A step performed during genomic testing, concluding the likelihood of a variant being associated with, and causative of, the clinical features (phenotype) in the patient. Classifications are benign, likely benign, variant of uncertain/unknown significance, likely pathogenic or pathogenic. |
| **Variant pathogenicity** | Likelihood that a variant is associated with, and causative of, the clinical features (phenotype) in the patient. |
| **Variant prioritisation** | A step performed during genomic testing, prioritising variants in genes more likely to be linked with the clinical features (phenotype) in the patient. |
| **Whole exome sequencing (WES)** | Determining the sequence of all the exons in a genome. |
| **Whole genome sequencing (WGS)** | Determining the sequence of all the DNA (coding and non-coding). |

# Section 1: Demographics

This section asks questions about you and your medical training, employment and areas of practice.

Note: as some demographic data could identify individual respondents in areas with few specialists, we will combine data for some analyses.

Words with a dotted underline and asterix* have a definition on roll-over or at the top of the question.

1. **What is your gender?**

| - Male - Female | - Other - Prefer not to answer |
| --- | --- |

1. **What is your age bracket?**

| - 24 or under - 25–34 | - 35–44 - 45–54 | - 55–64 - 65 or over |
| --- | --- | --- |

1. **Where are you located?** *Select one option.*

- Australia
- Other 🡪 *REDCap coding instruction go to SURVEY STOP “Thank you for your interest in our research but this survey is only for medical specialists currently practicing in Australia.”*
  1. Which state?

| - ACT - NSW | - NT - QLD | - SA - TAS | - VIC - WA |
| --- | --- | --- | --- |

1. **Do you see patients in your practice?** *This may be in a clinical or research setting.*

- Yes
- No 🡪 *REDCap coding instruction go to SURVEY STOP “Thank you for your interest in our research but this survey is only for medical specialists that see patients in their practice.”*

1. **What is your current level of specialty certification?** *Select all that apply, including options for dual trainees if applicable*

|  | **Basic trainee** | **Advanced trainee** | **Fellow** |
| --- | --- | --- | --- |
| Australasian College of Dermatologists (ACD) |  |  |  |
| Australasian College of Emergency Medicine (ACEM) |  |  |  |
| Australasian College of Anaesthetists (ANZCA) |  |  |  |
| Australasian College of Sport and Exercise Physicians (ACSEP) |  |  |  |
| Australian College of Rural and Remote Medicine (ACRRM) |  |  |  |
| College of Intensive Care Medicine of Australia and New Zealand (CICM) |  |  |  |
| Royal Australian and New Zealand College of Ophthalmologists (RANZCO) |  |  |  |
| Royal Australian and New Zealand College of Psychiatrists (RANZCP) |  |  |  |
| Royal Australian College of General Practitioners (RACGP) |  |  |  |
| Royal Australian College of Obstetricians & Gynaecologists (RANZCOG) |  |  |  |
| Royal Australasian College of Medical Administrators (RACMA) |  |  |  |
| Royal Australasian College of Physicians (RACP) |  |  |  |
| Royal Australasian College of Surgeons (RACS) |  |  |  |
| Royal Australasian College of Radiologists (RANZCR) |  |  |  |
| Royal College of Pathologists of Australasia (RCPA) |  |  |  |
| Other (please specify)…………………… |  |  |  |

1. **If you selected sub-specialist, what area is your sub-specialty training in? ……………………**
2. **In what year did you complete your medical degree (MBBS/MD)? ……………………**
3. **What medical specialty are you qualified for, accredited in or studying towards?**

*Please indicate your primary medical specialty. If you have dual training please select a second option, and even a third if you completed advanced training in another specialty.*

*If you are a sub-specialist in paediatrics, surgery or obstetrics and gynaecology, please select your specialty below and then choose from the list of sub-specialties that will appear, e.g., paediatric neurologist would select "Paediatrics" and then "Paediatric Neurology" from the list of sub-specialties.*

***Note:*** *there are separate studies for those whose primary specialty is clinical genetics, oncology, radiology, pathology and general practice, who will be contacted directly.*

*If your speciality is not listed, please select ‘Other’ and specify.*

| **Primary medical specialty** | **Secondary medical specialty** | **Tertiary medical specialty** |
| --- | --- | --- |
| Drop down list of below options | Drop down list of below options excluding answer for primary specialty*.* | Drop down list of below options excluding answers for primary and secondary specialty |

| - Addiction medicine - Anaesthesiology - Cardiology - Clinical genetics 🡪 *REDCap coding instruction go to SURVEY STOP* *“Thank you for your interest in our research but there is a separate survey for clinicians whose primary specialty is clinical genetics.”* - Clinical pharmacology - Dermatology - Emergency Medicine - Endocrinology - Gastroenterology and hepatology - General medicine - General practice 🡪 *REDCap coding instruction go to SURVEY STOP* *“Thank you for your interest in our research but there is a separate survey for clinicians whose primary specialty is general practice.”* | - Gerontology - Haematology - Immunology - Immunology and allergy - Infectious disease - Intensive Care - Medical administrator - Medical oncology 🡪 *REDCap coding instruction go to SURVEY STOP* *“Thank you for your interest in our research but there is a separate survey for clinicians whose primary specialty is oncology.”* - Nephrology - Neurology - Nuclear medicine - Obstetrics and Gynaecology 🡪 *REDCap branching: reveal* ***question 7a.*** - Ophthalmology - Pain medicine | - Palliative medicine - Pathology - Paediatrics 🡪 *REDCap branching: reveal* ***question 7b.*** - Public health medicine - Psychiatry - Radiology 🡪 *REDCap coding instruction go to SURVEY STOP* *“Thank you for your interest in our research but there is a separate survey for clinicians whose primary specialty is radiology.”* - Rehabilitation medicine - Respiratory and sleep medicine - Rheumatology - Sexual health medicine - Sport and exercise medicine - Surgery 🡪 *REDCap branching: reveal* ***question 7c.*** - Other (please specify)………………… |
| --- | --- | --- |

- 1. **If your specialty is Obstetrics and gynaecology, please indicate your sub-specialty.** *Select all that apply*

| - Gynaecological oncologist - Maternal-fetal medicine - Obstetrics and gynaecology - Obstetrics and gynaecological ultrasound | - Reproductive endocrinology and infertility - Urogynaecologist - Other (please specify) ………………… |
| --- | --- |

- 1. **If your specialty is paediatrics, please indicate your sub-specialty.** *Select all that apply.*

| - Clinical genetics - Community child health - General paediatrics - Neonatal and perinatal medicine - Paediatric cardiology - Paediatric clinical pharmacology - Paediatric emergency medicine - Paediatric endocrinology | - Paediatric gastroenterology and hepatology - Paediatric haematology - Paediatric immunology and allergy - Paediatric infectious diseases - Paediatric intensive care medicine - Paediatric medical oncology - Paediatric nephrology | - Paediatric neurology - Paediatric nuclear medicine - Paediatric palliative medicine - Paediatric rehabilitation medicine - Paediatric respiratory and sleep medicine - Paediatric rheumatology - Other (please specify)…………………… |
| --- | --- | --- |

- 1. **If your specialty is surgery, please indicate your sub-specialty.** *Select all that apply*

| - Cardio-thoracic surgery - General surgery - Neurosurgery - Orthopaedic surgery - Otolaryngology - Oral and maxillofacial surgery | - Paediatric surgery - Plastic surgery - Urology - Vascular surgery - Other (please specify) ………………… |
| --- | --- |

1. **Which categories of patients do you see?** *Select all that apply.*

- Pre-conception
- Pregnant
- Paediatric
- Adult
- Other (please specify)……………………

1. **Who is your main employer? *Select one option.*** *If you have more than one job, please indicate your employer for your primary specialty then secondary employer, etc. If your option is not listed, please tick other and specify below.*

*If you have two equal FTE appointments, please consider your clinical appointment as your primary role. If you have equal clinical roles across two employers, please select one.*

| **Employer for primary job** | **Employer for secondary job** | **Employer for tertiary job** |
| --- | --- | --- |
| Drop down list of below options | Drop down list of below options excluding answer for primary employer | Drop down list of below options excluding answers for primary and secondary employer |

- Public hospital or healthcare provider
- Private hospital or healthcare provider
- University or education provider, e.g., medical specialty College
- Research institute
- Community health centre
- Government
- Self-employed/ private practice
- Patient advocacy or support group
- Other (please specify)……………………
- Not currently practising in my medical specialty

1. **In the last 12 months, what was your main work location?** *Select one option.*

*If you have more than one job, please indicate the location for your primary specialty.*

*If you are unsure of your main work location, please refer to the Australia Bureau of Statistics remoteness classifications by clicking here.*

| - Major city (RA1) - Inner regional (RA2) - Outer regional (RA3) | - Remote (RA4) - Very remote (RA5) - Other (please specify)…………………… |
| --- | --- |

# Section 2: Genomics in Practice – current and future

We are gathering information about medical specialists’ genomics practice, both research and clinical. We will also ask you to reflect on your future practice and involvement in genomics.

Some questions may feel repetitive but the data will help workforce planning across all aspects of patient care, including outside the clinic, such as research, education, training and policy.

Your responses may cause some questions to appear/disappear. This is a normal function of this survey to keep it as short as possible

Note: words with a dotted underline and asterix* have a definition on roll-over or at the top of the question.

1. **Do clinical guidelines exist for genomic sequencing testing* in your specialty?** *These may be local, national or international*

*The below definition/s may help answer this question:*

*Genomic sequencing testing: a genomic test investigates many regions of the genome at once. For the purpose of this survey, genomic tests based on sequencing technologies are defined to include gene panels, whole exomes or whole genomes, but to exclude non-invasive prenatal testing using sequencing technologies*

- Yes 🡪 *REDCap branching: reveal* ***question a.***
- No
- Unsure

1. **If yes, who provides these guidelines?** *(e.g., RACP, HGSA, hospital etc.)* ………………
2. **Have you been involved in any genomic research projects in the last 5 years?**

- Yes
- No
- I would like to but have not had the opportunity
- Unsure

1. **If yes, what type of research project was it?** *Select all that apply.*

*The below definition/s may help answer this question:*

*ELSI: Ethical, legal and social implications*

- Laboratory
- Clinical
- Social science (ELSI*, education, policy, etc.)
- Bioinformatics
- Other (please specify)……………………

1. **Have you contacted your clinical genetics team or service in the last 12 months?**

- Yes 🡪 *REDCap branching: reveal* ***question a-b.***
- No 🡪 *REDCap branching: reveal* ***question c.***
- Unsure

1. **If yes, how frequently was this?** *Select one only*

| - Daily | - Weekly | - Monthly | - Quarterly | - Once or twice | - Unsure |
| --- | --- | --- | --- | --- | --- |

1. **Why did you contact your clinical genetics team or service?** *Select all that apply*
   - Information about a suspected genetic condition
   - Advice on what type of genetic or genomic test to order

- Advice on how to refer the patient to my clinical genetics team or service
- Assistance with genetic counselling before the test
- Assistance with genetic counselling after the test
  - Other (please specify)……………………

1. **Why haven’t you contacted your clinical genetics team or service?** *Select all that apply.*

- Genetics and genomics are not relevant to my practice
- I have not yet needed advice from a clinical genetics team or service in my practice
- I can manage my patients without advice from a clinical genetics service
- I’m not sure how to contact my clinical genetics team or service
- I do not have access to a clinical genetics team or service
- Other (please specify)……………………

**CHROMOSOMAL MICROARRAY**

1. **Did you order chromosomal microarray (microarray)* tests in the last 12 months as part of your clinical or research role?** *Select one option*

*The below definition/s may help answer this question:*

*Chromosomal microarray (microarray): a diagnostic genomic test to identify changes to copy number variants associated with disease.*

- Yes 🡪 *REDCap branching: reveal* ***questions a-c****.*
- No 🡪 *REDCap branching: reveal* ***questions d****.*

1. **If yes, how frequently did you order microarray tests in the last 12 months?**

| - Daily | - Weekly | - Monthly | - Quarterly | - Once or twice | - Unsure |
| --- | --- | --- | --- | --- | --- |

1. **How is/was this genomic testing funded?** *Select all that apply.*

| - Research grant - Institute/ hospital funding - State government funding - Medicare (national government funding) | - Patient funding - Other (please specify) ……………… - Unsure |
| --- | --- |

1. **How confident are you about the following aspects of microarray tests?** *Select a number to indicate how confident you feel, or tick N/A if you have not performed a task in your practice.*

*The below definition/s may help answer this question:*

*Variant of uncertain/unknown significance: a change in DNA sequence where it is unclear whether it is disease-causing.*

|  | **N/A** | **1** | **2** | **3** | **4** | **5** | **6** | **7** | **8** | **9** | **10** |
| --- | --- | --- | --- | --- | --- | --- | --- | --- | --- | --- | --- |
|  |  | **Not at all confident 🡪 Very confident** | | | | | | | | | |
| Understanding indications for microarray testing |  |  |  |  |  |  |  |  |  |  |  |
| Discussing microarray testing with families, e.g., technical aspects, limitations, variants of uncertain/unknown significance*, etc. |  |  |  |  |  |  |  |  |  |  |  |
| Facilitating patient informed consent for microarray, e.g., risks and benefits, incidental findings, impact on families, etc. |  |  |  |  |  |  |  |  |  |  |  |
| Understanding microarray reports |  |  |  |  |  |  |  |  |  |  |  |
| Verifying reports by checking literature and databases, e.g., [OMIM](https://www.omim.org/), [DECIPHER](https://decipher.sanger.ac.uk/), [gnomAD](http://gnomad.broadinstitute.org/)/[ExAC](http://exac.broadinstitute.org/) etc. |  |  |  |  |  |  |  |  |  |  |  |
| Discussing microarray results with patients/ families |  |  |  |  |  |  |  |  |  |  |  |

**Please provide more comments if you want to clarify**………………

1. **Why have you not ordered microarray tests in the last 12 months?** *Select all that apply*
   - I referred patients who required microarray testing to a clinical genetics team or other specialist service
   - I am unable to order a microarray test in my current role/ department, e.g., lack of access to testing or funding 🡪 *REDCap branching: reveal* ***question i****.*
   - I’m not sure how to order a microarray test
   - I’m not sure of the relevance of microarray to my practice
   - Microarray tests are not relevant to my practice
   - Other (please specify)……………………
     1. **If you are unable to order a microarray test, please explain why….**

**GENE PANEL TESTS**

1. **Did you order gene panel tests* in the last 12 months as part of your clinical or research role?** *Select one option*

*The below definition/s may help answer this question:*

*Gene panel test:* *a* *laboratory test to identify variants in several well characterised genes associated with a phenotype and clinical presentation*

- Yes 🡪 *REDCap branching: reveal* ***questions a-c****.*
- No 🡪 *REDCap branching: reveal* ***questions d****.*

.

1. **If yes, how frequently did you order gene panel tests in the last 12 months?**

| - Daily | - Weekly | - Monthly | - Quarterly | - Once or twice | - Unsure |
| --- | --- | --- | --- | --- | --- |

1. **How is/was this genomic testing funded?** *Select all that apply.*

| - Research grant - Institute/ hospital funding - State government funding - Medicare (national government funding) | - Patient funding - Other (please specify) ……………… - Unsure |
| --- | --- |

1. **What type of gene panel test did you order in the last 12 months?** *Select all that apply.*

| - Germline - Somatic - Unsure |
| --- |

1. **How confident are you about the following aspects of gene panel tests?** *Select a number to indicate how confident you feel, or tick N/A if you have not performed a task in your practice.*

*The below definition/s may help answer this question:*

*Variant of uncertain/unknown significance: a change in DNA sequence where it is unclear whether it is disease-causing.*

|  | **N/A** | **1** | **2** | **3** | **4** | **5** | **6** | **7** | **8** | **9** | **10** |
| --- | --- | --- | --- | --- | --- | --- | --- | --- | --- | --- | --- |
|  |  | **Not at all confident 🡪 Very confident** | | | | | | | | | |
| Understanding indications for gene panel testing |  |  |  |  |  |  |  |  |  |  |  |
| Discussing gene panel testing with families, e.g., technical aspects, limitations, variants of uncertain/unknown significance*, etc. |  |  |  |  |  |  |  |  |  |  |  |
| Facilitating patient informed consent for gene panel, e.g., risks and benefits, incidental findings, impact on families, etc. |  |  |  |  |  |  |  |  |  |  |  |
| Understanding gene panel reports |  |  |  |  |  |  |  |  |  |  |  |
| Verifying reports by checking literature and databases, e.g., [OMIM](https://www.omim.org/), [DECIPHER](https://decipher.sanger.ac.uk/), [gnomAD](http://gnomad.broadinstitute.org/)/[ExAC](http://exac.broadinstitute.org/) etc. |  |  |  |  |  |  |  |  |  |  |  |
| Discussing gene panel results with patients/ families |  |  |  |  |  |  |  |  |  |  |  |

**Please provide more comments if you want to clarify**………………

1. **Why have you not ordered gene panel tests in the last 12 months?** *Select all that apply*
   - I referred patients who required gene panel testing to a clinical genetics team or other specialist service
   - I am unable to order a gene panel test in my current role/ department, e.g., lack of access to testing or funding 🡪 REDCap branching: reveal question i.
   - I’m not sure how to order a gene panel test
   - I’m not sure of the relevance of gene panel tests to my practice
   - Gene panel tests are not relevant to my practice
   - Other (please specify)……………………
     1. **If you are unable to order a gene panel test, please explain why….**

**WHOLE EXOME/WHOLE GENOME SEQUENCING TESTS**

1. **Did you order whole exome* or whole genome** sequencing tests in the last 12 months as part of your clinical or research role?** *Select one option*

*The below definition/s may help answer this question:*

*Whole exome sequencing: the process of determining the sequence of all the exons in a genome.*

*Whole genome sequencing: the process of determining the sequence of all the DNA (coding and non-coding).*

- Yes 🡪 *REDCap branching: reveal* ***questions a-c****.*
- No 🡪 *REDCap branching: reveal* ***questions d****.*

1. **If yes, how frequently did you order whole exome/genome sequencing tests in the last 12 months?**

| - Daily | - Weekly | - Monthly | - Quarterly | - Once or twice | - Unsure |
| --- | --- | --- | --- | --- | --- |

1. **How is/was this genomic testing funded?** *Select all that apply.*

| - Research grant - Institute/ hospital funding - State government funding - Medicare (national government funding) | - Patient funding - Other (please specify) ……………… - Unsure |
| --- | --- |

1. **What type of whole exome/genome sequencing test did you order in the last 12 months?** *Select all that apply.*

| - Germline - Somatic - Unsure |
| --- |

1. **How confident are you about the following aspects of whole exome/genome sequencing tests?** *Select a number to indicate how confident you feel, or tick N/A if you have not performed a task in your practice.*

*The below definition/s may help answer this question:*

*Variant of uncertain/unknown significance: a change in DNA sequence where it is unclear whether it is disease-causing.*

|  | **N/A** | **1** | **2** | **3** | **4** | **5** | **6** | **7** | **8** | **9** | **10** |
| --- | --- | --- | --- | --- | --- | --- | --- | --- | --- | --- | --- |
|  |  | **Not at all confident 🡪 Very confident** | | | | | | | | | |
| Understanding indications for whole exome/genome sequencing testing |  |  |  |  |  |  |  |  |  |  |  |
| Discussing whole exome/genome sequencing testing with families, e.g., technical aspects, limitations, variants of uncertain/unknown significance*, etc. |  |  |  |  |  |  |  |  |  |  |  |
| Facilitating patient informed consent for whole exome/genome sequencing, e.g., risks and benefits, incidental findings, impact on families, etc. |  |  |  |  |  |  |  |  |  |  |  |
| Understanding whole exome/genome sequencing reports |  |  |  |  |  |  |  |  |  |  |  |
| Verifying reports by checking literature and databases, e.g., [OMIM](https://www.omim.org/), [DECIPHER](https://decipher.sanger.ac.uk/), [gnomAD](http://gnomad.broadinstitute.org/)/[ExAC](http://exac.broadinstitute.org/) etc. |  |  |  |  |  |  |  |  |  |  |  |
| Discussing whole exome/genome sequencing results with patients/ families |  |  |  |  |  |  |  |  |  |  |  |

**Please provide more comments if you want to clarify**………………

1. **Why have you not ordered whole exome/genome sequencing tests in the last 12 months?** *Select all that apply*
   - I referred patients who required whole exome/genome sequencing testing to a clinical genetics team or other specialist service
   - I am unable to order a whole exome/genome sequencing test in my current role/ department, e.g., lack of access to testing or funding 🡪 REDCap branching: reveal question i.
   - I’m not sure how to order a whole exome/genome sequencing test
   - I’m not sure of the relevance of whole exome/genome tests to my practice
   - whole exome/genome sequencing tests are not relevant to my practice
   - Other (please specify)……………………
     1. **If you are unable to order a whole exome/genome sequencing test, please explain why….**

The next question will help map the future genomic practices of medical specialists in Australia and develop education to suit these needs. It doesn't matter if you do or don't know much about these areas, or don't incorporate them into your practice at the moment; your opinions and views are valuable to us.

1. **Below is a list of some of the steps involved in genomic sequencing testing* from pre-test to post-test. Please indicate which steps you currently perform and which ones you expect to perform in the future if you had adequate education, training and support.** *Please select one option per row.*

*The below definition/s may help answer this question:*

*Genomic sequencing testing: a genomic test investigates many regions of the genome at once. For the purpose of this survey, genomic tests based on sequencing technologies are defined to include gene panels, whole exomes or whole genomes, but to exclude non-invasive prenatal testing using sequencing technologies.*

*Variant of uncertain/unknown significance: A change in DNA sequence where it is unclear whether it is disease-causing.*

*Variant pathogenicity: Likelihood that a variant is associated with, and causative of, the clinical features (phenotype) in the patient.*

*Variant prioritisation: A step performed during genomic testing, prioritising variants in genes more likely to be linked with the clinical features (phenotype) in the patient.*

*Variant classification: A step performed during genomic testing, concluding the likelihood of a variant being associated with, and causative of, the clinical features (phenotype) in the patient. Classifications are benign, likely benign, variant of uncertain/unknown significance, likely pathogenic or pathogenic.*

*Note: this question does NOT relate to microarray or gene panel tests. We are only asking about whole exome/genome sequencing tests in the question.*

*We appreciate not all genomic sequencing testing follows the same process. These steps are just a guide; please use the Comment box below to describe other steps you perform or expect to perform.*

|  | **Currently perform and expect to continue** | **Currently perform and do not expect to continue** | **Do not currently perform but expect to** | **Do not currently perform and do not expect to** | **Unsure** | **N/A** |
| --- | --- | --- | --- | --- | --- | --- |
| **Pre-test** | | | | | | |
| Eliciting information about genetic conditions as part of a family or medical history |  |  |  |  |  |  |
| Identifying a patient suitable for a genomic test |  |  |  |  |  |  |
| Pre-test counselling to assist in making an informed decision, e.g., genetics, test limitations, variants of uncertain/unknown significance**, incidental/secondary findings, unexpected non-paternity or consanguinity |  |  |  |  |  |  |
| Ordering a genomic test for a patient |  |  |  |  |  |  |
| **Test** | | | | | | |
| Attending multidisciplinary team meeting to discuss the genomic test (e.g., intake meeting) |  |  |  |  |  |  |
| Assisting the lab to narrow down the genes of interest (creating a gene list to prioritise variant analysis) |  |  |  |  |  |  |
| Providing phenotypic information to the lab to prioritise variant analysis |  |  |  |  |  |  |
| Laboratory and bioinformatics testing processes |  |  |  |  |  |  |
| Searching the literature and databases for evidence of variant pathogenicity*** |  |  |  |  |  |  |
| Attending a multidisciplinary team meeting to discuss variant prioritisation****, interpretation and classification***** |  |  |  |  |  |  |
| **Post-test** | | | | | | |
| Provide test results to patients/ families |  |  |  |  |  |  |
| Provide genetic counselling to patients/families, e.g., explain variants of uncertain/unknown significance**, incidental/secondary findings, unexpected non-paternity or consanguinity |  |  |  |  |  |  |
| Organising/ referring for further testing of family members if required, e.g., cascade testing or segregation studies |  |  |  |  |  |  |
| Ongoing management of the patient, e.g., clarify recurrence risk and discuss reproductive planning options |  |  |  |  |  |  |
| Post-test follow up of patient to check understanding of result/ ask any additional questions |  |  |  |  |  |  |
| Other (please specify)…………………… |  |  |  |  |  |  |

**Please explain why you do not expect to perform the selected steps/s:** *REDCap coding🡪 pipe in responses*…………….

**Comment (optional)**…………….

1. **What is/would be your preferred model for delivering a genomic sequencing test* in your clinical practice, assuming you have appropriate education, training and funding?** *Select one option for inpatient and one option for outpatient.*

*You may have more than one preference; please indicate your* ***FIRST*** *preference. If you have more than one specialty, please respond for your primary specialty. Your reasoning and preferences can be described in the Comments box.*

*Note: this question does NOT relate to microarray or gene panel tests. We are only asking about whole exome/genome sequencing tests in the question.*

*The below definition/s may help answer this question:*

*Genomic sequencing testing: A genomic test investigates many regions of the genome at once. For the purpose of this survey, genomic tests based on sequencing technologies are defined to include gene panels, whole exomes or whole genomes, but to exclude non-invasive prenatal testing using sequencing technologies.*

|  | **INPATIENT** | **OUTPATIENT** |
| --- | --- | --- |
| You initiate testing and discuss results with patients/families |  |  |
| You initiate testing and discuss results with patients/families, with support from a clinical genetics team as needed 🡪 *REDCap branching: reveal* ***question i.*** |  |  |
| You refer to a clinical genetics team to initiate testing and discuss results with patients/families |  |  |
| You do not see, and do not expect to see, patients who would benefit from genomic testing |  |  |
| Unsure at this stage |  |  |
| Other (please specify, e.g., preference for inpatient vs outpatient setting )…………………… |  |  |

1. **If support is needed, please indicate which areas might be most helpful.** *Select as many as apply*
   - Advice on whether test is appropriate

- Pre-test counselling
- Consent
- Interpreting results
- Discussing results with families
  - Follow-up genetic counselling of family
  - Other (please specify)……………………

**Please provide more comments if you want to clarify,** *e.g., details of support, discussion across disciplines for support…………………….*

**What factors influence your preferred model of genomic test delivery?** *e.g., funding*, *impact of test result, patient characteristics, etc. …………………………………………*

1. **Below is a list of ways genomic sequencing tests* and other genomic tests** can be initiated and discussed with patients. Please indicate which currently occur in your practice and/ or you believe will occur more frequently in the next five years.** *Select all that apply in each column*

*The below definition/s may help answer this question:*

*Genomic sequencing testing: A genomic test investigates many regions of the genome at once. For the purpose of this survey, genomic tests based on sequencing technologies are defined to include gene panels, whole exomes or whole genomes, but to exclude non-invasive prenatal testing using sequencing technologies.*

*Other genomic tests: A genomic test investigates many genes at once. For the purpose of this survey, genomic tests not based on sequencing technologies are defined to include non-invasive prenatal tests and chromosomal microarrays.*

*Direct to consumer/personal genomic tests and/or online DNA testing: This may be any query, e.g., general questions, testing for their condition, results from online DNA testing that patients bring to clinic and/or medical implications.*

|  | **Currently occurs and will not change** | **Currently occurs and will occur more frequently in next 5 years** | **Does not currently occur but will occur more frequently in next 5 years** | **Does not currently occur and will not change** | **Unsure** |
| --- | --- | --- | --- | --- | --- |
| Clinicians request genetic or genomic tests to aid in diagnosis/ prognosis/ treatment/ ongoing management |  |  |  |  |  |
| Clinicians request pharmacogenomic tests to aid in treatment |  |  |  |  |  |
| Clinicians refer for genetic or genomic tests to aid in diagnosis/ prognosis/ treatment/ ongoing management, including pharmacogenomic tests |  |  |  |  |  |
| Patients/ families ask about genetic or genomic tests to aid in diagnosis/ prognosis/ treatment/ ongoing management |  |  |  |  |  |
| Patients/ families ask about direct to consumer/personal genomic tests and/or online DNA testing***, such as SmartDNA or 23&Me |  |  |  |  |  |

**Do you think genomics will impact your practice in the next 2 years?**

Yes 🡪 *REDCap branching: reveal* ***question b.***

No

Unsure

- 1. **Why?................**
  2. **What areas will be impacted?** *Select all that apply.*
- The way I practice medicine
- My workload
- Patient management
- Other (please specify)……………………

**Please provide more comments if you want to clarify**………………

1. **Do you feel prepared to use genomic sequencing testing* in your practice?**

*The below definition/s may help answer this question:*

*Genomic sequencing testing: A genomic test investigates many regions of the genome at once. For the purpose of this survey, genomic tests based on sequencing technologies are defined to include gene panels, whole exomes or whole genomes, but to exclude non-invasive prenatal testing using sequencing technologies.*

- Yes
- No
- Unsure
  1. **Please explain your response.** *E.g., what might need to change to help you feel prepared?*

# Section 3: Education and training – current and future

The following questions relate to previous and future professional development. Some questions and responses may seem repetitive, however these data will assist with planning workforce education and training, more generally and specifically in genomics.

Remember words with a dotted underline and asterix* have a definition on roll-over or at the top of the question.

1. **How confident are you in your….** *Select a number to indicate how confident you feel*

|  | **Unsure** | **1** | **2** | **3** | **4** | **5** | **6** | **7** | **8** | **9** | **10** |
| --- | --- | --- | --- | --- | --- | --- | --- | --- | --- | --- | --- |
|  |  | **Not at all confident 🡪 Very confident** | | | | | | | | | |
| Knowledge about genomics |  |  |  |  |  |  |  |  |  |  |  |
| Ability to elicit information about genetic conditions as part of a family or medical history |  |  |  |  |  |  |  |  |  |  |  |
| Ability to explain genomic concepts to patients |  |  |  |  |  |  |  |  |  |  |  |
| Ability to make decisions based on genomic information |  |  |  |  |  |  |  |  |  |  |  |

1. **What would help improve your confidence?...................**
2. **Would improving your knowledge of genomic medicine alter your practice?**

- Yes
- No
- Unsure

1. **If yes, please explain how……………………**
2. **If no, please explain why not and/or what factors may alter your practice……………………**
3. **Have you ATTENDED any professional development education or training around genomics in the *past year*, such as lectures, seminars or workshops, either in person or online?**

- Yes
- No
  1. **If yes, was this:**

*The below definition/s may help answer this question:*

*MOOC: massive, open, online course, such as Coursera or Future Learn*

- In-house (internal) program/s
- External program/s
- Online training (webinar, MOOC,* *etc*.)
- Other (please specify)……………………

1. **Have you PROVIDED any professional development education or training around genomics in the *past year*, such as lectures, seminars or workshops, either in person or online?**

- Yes
- No
  1. **If yes, was this:**
- In-house (internal) program/s
- External program/s
- Online training (webinar, MOOC,* *etc*.)
- Other (please specify)……………………

1. **Who should be responsible for updating medical specialists about genomics?** *Select all that apply.*

*REDCap coding instruction: randomly sort options*

- Universities
- Medical colleges
- Australian Medical Council
- Professional education providers, e.g., Centre for Genetics Education (<http://www.genetics.edu.au/>)
- National/ international genetic societies, e.g., Human Genetics Society of Australasia (<https://www.hgsa.org.au/>), American College of Medical Genetics (<https://www.acmg.net/>)
- State/ national genomic alliances, e.g., Australian Genomic Health Alliance (<https://www.australiangenomics.org.au/>), Queensland Genomic Health Alliance (<https://www.qgha.org/>)
- Medical Research Institutes with expertise in genomics, e.g., Garvan Institute of Medical Research (<https://www.garvan.org.au/>)
- Biotechnology companies
- Clinical genetic services
- Genetic/ genomic NATA laboratories or other pathology services
- Hospitals
- Specialists should update themselves
- Other (please specify)……………………

1. **If you selected other, please specify……………………**
2. **Below is a list of activities that can be used to keep up to date with, or learn new skills in, genomic medicine. Please indicate which activities you currently use and/or would prefer to use to keep up to date with, or learn new skills in, genomic medicine.** *Select one option per row.*

*The below definition/s may help answer this question:*

*MOOC****:*** *Massive, open, online course, such as Coursera or Future Learn.*

|  | **Currently use and will continue to use** | **Currently use but would prefer not to use** | **Do not currently use but would prefer to use** | **Do not currently use and would not use** | **Unsure** | **N/A** |
| --- | --- | --- | --- | --- | --- | --- |
| Certification/fellowship activities |  |  |  |  |  |  |
| CPD/CME activities |  |  |  |  |  |  |
| Time in a service or laboratory with genomics expertise, e.g., traineeship, immersion, observership |  |  |  |  |  |  |
| Genomic research project, e.g., collaboration with a research laboratory |  |  |  |  |  |  |
| Multidisciplinary meetings |  |  |  |  |  |  |
| Internal workplace specialty seminars, conferences or similar |  |  |  |  |  |  |
| Internal workplace genetic or genomic seminars, conferences, etc. |  |  |  |  |  |  |
| External specialty seminars, conferences, etc. |  |  |  |  |  |  |
| External genetic or genomic seminars, conferences, etc. |  |  |  |  |  |  |
| Online webinars, courses, MOOCs*, etc. |  |  |  |  |  |  |
| Reading specialty texts (journals, papers, books, etc.) |  |  |  |  |  |  |
| Study days at place of employment |  |  |  |  |  |  |
| Small group tutorials |  |  |  |  |  |  |
| Departmental presentations |  |  |  |  |  |  |
| Consult colleagues and peer |  |  |  |  |  |  |
| Mass media (TV, newspapers) |  |  |  |  |  |  |
| Social media, e.g., twitter journal club |  |  |  |  |  |  |
| Other (please specify)…………………… |  |  |  |  |  |  |

1. **Below is a list of topics in genomic medicine. Please indicate which you have learnt about and which you want to in the future.** *Select one option per row.*

*The below definition/s may help answer this question:*

*Variant of uncertain/unknown significance****:*** *A change in DNA sequence where it is unclear whether it is disease-causing.*

| **Topics** | **Have learnt about and want more** | **Have learnt about and do not want more** | **Have not learnt about but want to** | **Have not learnt about and do not want to** | **Unsure** | **N/A** |
| --- | --- | --- | --- | --- | --- | --- |
| **Genetics/ genomics knowledge:** | | | | | | |
| Basic concepts |  |  |  |  |  |  |
| Disorders and diseases |  |  |  |  |  |  |
| Current applications in genomic medicine |  |  |  |  |  |  |
| Emerging applications in genomic medicine |  |  |  |  |  |  |
| **Genetic/ genomic testing and technologies:** | | | | | |  |
| Different types of genetic tests, e.g., microarray, single gene tests |  |  |  |  |  |  |
| Different types of genomic tests, e.g., panels, whole exome/genome sequencing |  |  |  |  |  |  |
| Different applications of somatic genomic tests |  |  |  |  |  |  |
| Different applications of germline genomic tests |  |  |  |  |  |  |
| Clinical utility of tests, e.g., diagnosis/ prognosis/ treatment/ ongoing management, including pharmacogenomics |  |  |  |  |  |  |
| Classification of genomic data during testing |  |  |  |  |  |  |
| Limitations of testing, e.g., what types of mutations are not detected by tests |  |  |  |  |  |  |
| **Pre or post-test aspects:** | | | | | | |
| Recognising patients who may benefit from genomic testing |  |  |  |  |  |  |
| Communication skills with patients |  |  |  |  |  |  |
| Performing genetic risk assessments i.e., family health history risk assessments |  |  |  |  |  |  |
| Referring appropriately for a genomic test |  |  |  |  |  |  |
| Requesting a genomic test for a patient, e.g., pathology form, blood tubes, consent, etc. |  |  |  |  |  |  |
| Interpreting genomic test results |  |  |  |  |  |  |
| Identifying additional family members who may benefit from genomics testing (cascade testing) |  |  |  |  |  |  |

| **Topics** | **Have learnt about and want more** | | **Have learnt about and do not want more** | | **Have not learnt about but want to** | | **Have not learnt about and do not want to** | | **Unsure** | | **N/A** |
| --- | --- | --- | --- | --- | --- | --- | --- | --- | --- | --- | --- |
| **Ethical, legal, and social implications (ELSI) of genetic/genomic testing:** | | | | | | | | | | |  |
| Ethical implications, e.g., incidental findings, variants of uncertain/unknown significance*, etc. | |  | |  | |  | |  | |  |  |
| Legal implications, e.g., data sharing, privacy, confidentiality, insurance, etc. | |  | |  | |  | |  | |  |  |
| Psychosocial implications, e.g., family communication of information, reproductive options, linking patients to support groups, etc. | |  | |  | |  | |  | |  |  |
| **Other (please specify)……………………** | |  | |  | |  | |  | |  |  |

**Please list any other genomics topics on which you would like to receive education ……………………**

# Thank you

We are interested in speaking with some respondents further to speak about the above topics in more depth. If you are interested in being contacted for a phone interview at a mutually convenient time, please provide the best contact details for yourself (email or telephone) and one of our researchers will follow up.

If you wish to receive a copy of the study results, please provide your name and email address below**.** *Please note, if you provide your name and contact details, we will not use this information for research purposes. It will only be used to send you a copy of the results.*

***Thank you for taking the time to complete this survey. Your responses are invaluable in shaping the future of the profession.***

***Please distribute the survey link to other medical specialists in your network:***

***Don’t forget to click ‘Submit’ or your responses will not be included in our research.***

[SUBMIT](http://www.genioz.net.au/page/start-survey/)

*Should you require any further information, or have any concerns, please do not hesitate to contact
Dr Amy Nisselle, Specialist Project Officer, Australian Genomics Health Alliance
(T 03 9936 6340; E* [*amy.nisselle@mcri.edu.au*](mailto:amy.nisselle@mcri.edu.au)*).*
